# *In vitro* potentiation of tetracyclines in *Pseudomonas aeruginosa* by RW01, a new cyclic peptide

**DOI:** 10.1128/aac.01459-24

**Published:** 2024-12-23

**Authors:** Natalia Roson-Calero, María A. Gomis Font, Albert Ruiz-Soriano, Xavier Just-Baringo, María Eugenia Pachón-Ibáñez, J. Pablo Salvador, M. Pilar Marco, Ernest Giralt, Antonio Oliver, Clara Ballesté-Delpierre, Jordi Vila

**Affiliations:** 1 Barcelona Institute for Global Health (ISGlobal) 310844 , Barcelona, Spain; 2 Department of Basic Clinical Practice, School of Medicine, University of Barcelona 368895 , Barcelona, Spain; 3 CIBER de Enfermedades Infecciosas (CIBERINFEC), Instituto de Salud Carlos III 38176 , Madrid, Spain; 4 Department of Microbiology, Hospital Universitario Son Espases, Health Research Institute of the Balearic Islands (IdISBa) 375118 , Palma de Mallorca, Spain; 5 Laboratori de Química Orgànica, Facultat de Farmàcia, Universitat de Barcelona 73070 , Barcelona, Spain; 6 Clinical Unit of Infectious Diseases, Microbiology and Parasitology, Institute of Biomedicine of Seville (IBiS), Virgen del Rocio University Hospital/CSIC/University of Seville 16778 , Seville, Spain; 7 Nanobiotechnology for Diagnostics (Nb4D), Department of Chemical and Biomolecular Nanotechnology, Institute for Advanced Architecture of Catalonia (IQAC) of the Spanish Council for Scientific Research (CSIC) 441673 , Barcelona, Spain; 8 Centro de Investigación Biomédica en Red Bioingeniería, Biomateriales y Nanomedicina (CIBER_BBN) 468627 , Madrid, Spain; 9 Institute for Research in Biomedicine (IRB Barcelona), Barcelona Institute of Science and Technology 518635 , Barcelona, Spain; 10 Department of Inorganic and Organic Chemistry, University of Barcelona 602771 , Barcelona, Spain; 11 Department of Clinical Microbiology, Biomedical Diagnostic Center, Hospital Clinic 571524 , Barcelona, Spain; Columbia University Irving Medical Center, New York, New York, USA

**Keywords:** *Pseudomonas aeruginosa*, cyclic peptides, adjuvants, membrane disruption, antimicrobial resistance

## Abstract

The pipeline for new drugs against multidrug-resistant *Pseudomonas aeruginosa* remains limited, highlighting the urgent need for innovative treatments. New strategies, such as membrane-targeting molecules acting as adjuvants, aim to enhance antibiotic effectiveness and combat resistance. RW01, a cyclic peptide with low antimicrobial activity, was selected as an adjuvant to enhance drug efficacy through membrane permeabilization. RW01’s activity was evaluated via antimicrobial susceptibility testing in combination with existing antibiotics on 10 *P. aeruginosa* strains and analog synthesis. Synergy was assessed using checkerboard assays, and one-step mutants were generated to identify altered pathways through whole-genome sequencing and variant analysis. Permeabilizing activity was studied using flow cytometry and real-time fluorescence measurement. *In vivo* toxicity was assessed in female C57BL/6J mice, and possible interaction with mouse serum was also evaluated. Susceptibility testing revealed specific synergy with tetracyclines, with up to a 16-fold reduction in minimum inhibitory concentrations. Sequencing revealed that resistance to the RW01-minocycline combination involved mutations in the *pmrB* gene, affecting outer membrane lipopolysaccharide composition. This was further confirmed by the identification of cross-resistance to colistin in these mutants. RW01 reduced the mutant prevention concentration of minocycline from 64 to 8 mg/L. RW01 was demonstrated to enhance membrane permeabilization and therefore minocycline uptake with statistical significance. Synthetic derivatives of RW01 showed a complete loss of activity, highlighting the importance of RW01’s D-proline(NH2) residue. No acute or cumulative *in vivo* toxicity was observed in mice. These findings suggest that RW01 could revitalize obsolete antimicrobials and potentially expand therapeutic options against multidrug-resistant *P. aeruginosa*.

## INTRODUCTION

Multidrug-resistant (MDR) *Pseudomonas aeruginosa* remains one of the most concerning pathogens, leading to its inclusion in the World Health Organization’s Bacterial Priority Pathogen List 2024 (1). The critical challenge in treating infections caused by *P. aeruginosa* is its worrying ability to acquire and develop high levels of antimicrobial resistance (AMR) (2, 3), significantly elevating the risk of mortality associated with these infections (4). In 2019, *P. aeruginosa* was among the six leading pathogens responsible for the highest number of deaths, all together accounting for 929,000 of the 1.27 million deaths attributable to AMR and 3.57 million deaths associated with AMR (5). Its lower baseline susceptibility to antibiotics, compared to other Gram-negative pathogens, is likely driven by several key mechanisms like production of inducible AmpC cephalosporinase, constitutive or inducible expression of Mex-type RND-efflux pumps, or reduced outer membrane permeability (6, 7). Mutations in repressor genes like *mexR* or *nalC* commonly lead to the overexpression of these RND-efflux pumps, widening the resistance spectrum of *P. aeruginosa*, with such metabolic flexibility given by its large genome that even its fitness remains unaffected in the process (8, 9). For such reasons, *P. aeruginosa* is a major pathogen in healthcare environments, especially affecting immunocompromised patients, and its ability to spread via medical equipment or patient cross-contamination presents a serious threat to patient safety (9, 10).

The development of new antibiotics against MDR Gram-negative bacteria faces continuous challenges, resulting in only a limited number of cutting-edge antibiotics being introduced into clinical practice, while current treatment options are rapidly becoming obsolete. Reviewing the pipeline of new antimicrobial agents, studies show that there is no new in-class antimicrobial option aimed to cover multidrug-resistant *P. aeruginosa* (11). In view of the current unmet medical need for new drugs to treat drug-resistant *P*. aeruginosa, new strategies seem to appear to help fill this gap. Some of the strategies that seek to establish a foothold in the field are bacteriophage therapy, nanoparticles/nanomaterials, monoclonal antibodies, silencing RNA, or antimicrobial peptides (AMPs), among others (12, 13). The current clinical alternative pipeline for *P. aeruginosa* infections, including the ones associated with cystic fibrosis, is limited to three phage products named AP-PA02, YPT-01, and BX004-A, which are being evaluated in phase 1/phase 2 clinical trials (11). Nonetheless, parallel strategies involving the use of non-therapeutic molecules as boosting adjuvants to restore the efficacy of currently prescribed antibiotics, which are losing utility due to AMR, are also emerging (14–16).

Combination therapies are a common strategy, particularly for difficult-to-treat infections, to ensure broad coverage, reduce resistance rate and toxicity, and achieve synergistic effects. Synergy in antibiotic/adjuvant combinations frequently involves targeting different steps in metabolic pathways, enhancing drug uptake, or inhibiting enzymes, thereby increasing the effectiveness of the treatment. Main examples of such non-therapeutic boosting adjuvants are resistance-mechanism inhibitors and include β-lactamase inhibitors, efflux pump inhibitors, and permeability enhancers (16). An example of a recent adjuvant making it to clinical trial phase and being efficient against carbapenem-resistant *P. aeruginosa* is Sinovent’s XNW-4107, a novel β-lactamase inhibitor, named funobactam, thought to be combined with imipenem (11, 17, 18).

Deepening into permeabilizing adjuvants, membrane-targeting AMPs represent a promising strategy as boosters of antimicrobial uptake and are typically inspired by the naturally occurring AMPs (15, 19, 20). AMPs are generally oligopeptides with a length that varies from 5 to 100 amino acids and a wide range of targeted organisms (21). Unlike most antibiotics that target specific cellular processes, AMPs mainly target the lipopolysaccharide (LPS) layer of the cell’s outer membrane, a feature crucial for permeability enhancement (22). In this study, we investigate the potential of a synthetic cyclic peptide, termed RW01, as an effective antibiotic adjuvant candidate. We propose that RW01 may help in preventing the development of resistant mutants, enhancing the activity of existing antibiotics, and limiting *in vivo* toxicity.

## MATERIALS AND METHODS

### RW01 compound structure and analog synthesis

**Fig 1:**
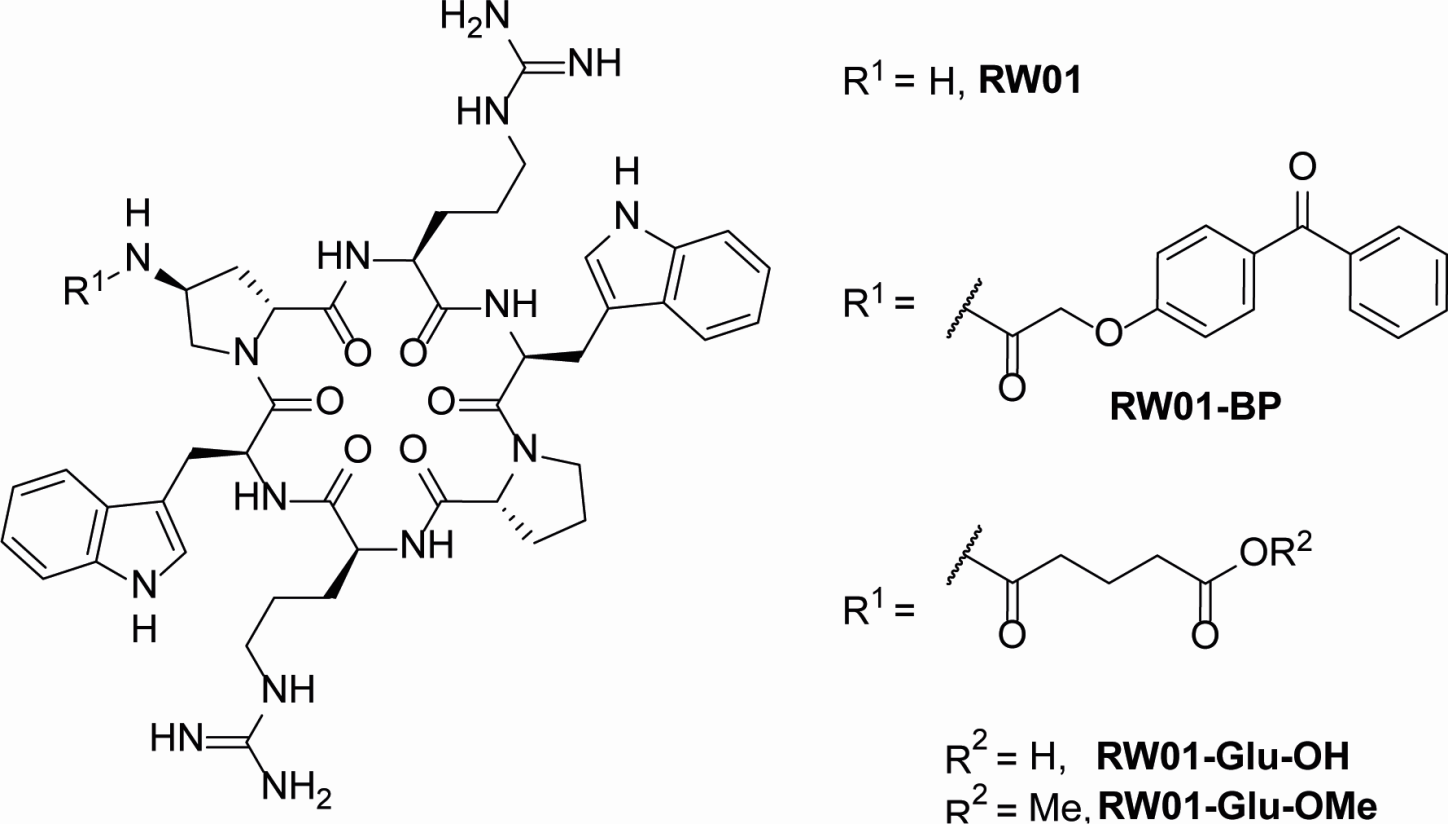
Chemical structure of RW01 cyclic peptide and its chemical analogs.

Based on previous studies, RW01 peptide was selected among a library of synthetic cyclic peptides (data not shown). The structure of the RW01 cyclic peptide consisted of six amino acids, two tryptophan residues, two D-proline (D-Pro), one modified with an amine group [D-Pro(NH_2_)], and two arginines (Arg). All together they form the cyclic structure &Trp-D-Pro(NH_2_)-Arg-Trp-D-Pro-Arg&. RW01 used in this study was commercially synthesized (GenicBio Limited, Shanghai, China), and its composition and purity were verified by mass spectrometry and high-performance liquid chromatography. RW01 was dissolved in Milli-Q distilled water. For the exploration of RW01’s activity and mode of action, different peptide analogs were synthesized using the free amine group in one of the D-Pro residues as handle. Three analogs were prepared, forming the corresponding amides by condensation with 2-(4-benzoylphenoxy)acetic acid (RW01-BP), glutaric anhydride (RW01-Glu-OH), and glutaric acid monomethyl ester chloride (RW01-Glu-OMe). All structures are represented in Fig. 1. Synthetic procedures, purification protocols, and usage of solvents/reagents are detailed in the Supplemental Information section.

### Strain selection and characterization

A collection of 10 previously characterized bacterial strains of *P. aeruginosa* were selected. The collection consisted of four clinical strains (23), wild-type PAO1 and PAOUW, and derivate mutants (24–26). Strain characteristics and specific mutations are shown in Table 1.

**TABLE 1:** Profile and characteristics of bacterial strains included in this study^*a*^

Strain	Resistance mechanism	Mutation	Characteristics
41A	–	–	Wild-type clinical isolate (23)
41B	Overexpression of *mexY.* *oprD* loss or decreased expression.	Deletion in *mexZ* (C_595_–C_605_). Insertions and deletions.	Isolated after treatment with meropenem (23)
125A	–	–	Wild-type clinical isolate (23)
125B	Overexpression of *mexB*	Mutation in *mexR* (MexR T98I)	Isolated after treatment with ceftazidime and meropenem (23)
PAO1	–	–	Wild-type reference strain (24, 25)
PAO∆*oprM*	–	**∆***oprM*::lox	*oprM*-deficient PAO1, gene that encodes the outer membrane protein component of MexAB-OprM and MexXY-OprM efflux pumps (24)
PAO∆*mexR*	Overexpression of *mexB*	**∆***mexR*::lox	*mexR*-deficient PAO1, gene that encodes the negative regulator of MexAB-OprM (24)
PAO∆*mexZ*	Overexpression of *mexY*	**∆***mexZ*::lox	*mexZ*-deficient PAO1, gene that encodes the negative regulator of MexXY-OprM (24)
PAOUW	–	–	Wild-type reference strain (26)
PAOUW∆*oprF*	–	**∆** *oprF*	*oprF*-deficient PAOUW, gene that encodes the major outer membrane protein OprF (26)

^a^ –, not applicable/available.

### Antimicrobial susceptibility testing

The minimum inhibitory concentration (MIC) of each antibiotic was determined as the lowest concentration that completely inhibited visible bacterial growth after 18–22 h of incubation at 37°C. Antimicrobial susceptibility testing was conducted according to Clinical and Laboratory Standards Institute (CLSI) guidelines using the microdilution technique in 96-well microtiter plates. The growth medium used for MIC determination was commercial BD Phoenix AST Broth (Becton Dickinson, New Jersey, USA) (27). Antimicrobials selected for testing in *P. aeruginosa* included aztreonam, cefepime, ceftazidime, chloramphenicol, doxycycline, imipenem, levofloxacin, meropenem, minocycline, netilmicin, norfloxacin, tetracycline, and tigecycline. MIC for RW01 was also tested. Using the RW01 MIC for the wild-type PAO1 reference strain as a baseline, all assays were performed at a concentration equal to one-fifth of the MIC (0.2× MIC). In this case, a combination of peptide-antibiotic was tested at a stable concentration of 100 mg/L of RW01 and serial dilutions of each antibiotic. Three biological replicates of each MIC were obtained. Parallelly, potential RW01 interaction with mice non-inactivated serum was determined by performing antimicrobial susceptibility assays to obtain the MIC of minocycline and doxycycline ±RW01 in the presence of 25% of a commercial mice serum from inbreed, C57BL/6RJ male mice (Tebubio, Yvelines, France). Wild-type PAO1 reference strain was used for MIC determination.

### Checkerboards

To assess the *in vitro* interaction between RW01 and tetracyclines, the checkerboard assay method was performed in 96-well microtiter plates using strains PAO1 and PAOUW. In the same way as for MIC determination, BD Phoenix AST Broth (Becton Dickinson) medium was used for bacterial growth. Minocycline and tigecycline combined with RW01 were the agents selected for the assay. A column and a row of wells in the checkerboard plate were reserved for controls of each antimicrobial individually to ensure the proper concentration of the agents. Positive and negative controls were also included. Wells in rows contained serial dilutions of RW01 starting from 512 mg/L, while those in columns varied in concentrations of the antibiotic. Inoculum at ~5 × 10⁵ CFU/mL, antibiotic, RW01, and AST broth were added to a final volume of 200 µL. Plates were incubated at 37°C for 18–22 h under aerobic conditions. Three biological replicates were obtained. Fractional inhibitory concentration index (FICI) is defined as the summatory of fractional inhibitory concentrations (FICs) from compound A: RW01, and compound B: minocycline/tigecycline. FICs were calculated as:


$$\begin{array}{l} FIC\;of\;RW01\;\left(FIC_{RW01}\right)=\frac{MIC\;of\;RW01\;in\;combination}{MIC\;of\;RW01\;alone},\\ FIC\;of\;antibiotic\;\left(FIC_{ATB}\right)=\frac{MIC\;of\;ATB\;in\;combination}{MIC\;of\;ATB\;alone},\\ FICI=FIC_{RW01}\;+FIC_{ATB}. \end{array}$$

FICI was interpreted as follows: synergistic effect if FICI is ≤0.5; additive effect if 0.5 < FICI ≤ 1.0; indifferent effect if 1.0 < FICI ≤ 2.0; and finally, antagonistic effect if FICI is >2.0. The average FICI considering the antibiotic MIC (no growth in any of the replicates) at different concentrations of RW01 was calculated (28).

### One-step mutant generation and resistant population analysis

Selection of resistant mutants was performed for minocycline with and without RW01 in three independent replicates for wild-type *P. aeruginosa* reference strain PAO1. Tubes containing Mueller-Hinton broth (MHB) were inoculated and incubated for 24 h at 37°C. Afterward, 1/100 dilution of each tube was incubated at 37°C in MHB to late-exponential growth phase, measured by spectrophotometry at OD_600_. Serial dilutions of PAO1 were then performed and plated in Mueller-Hinton agar (MHA) containing different concentrations of minocycline, alone and in the presence of RW01 at 100 mg/L. In-plate concentrations ranged from 0.125 to 128 mg/L (log 2 scale) of minocycline. A control plate without antibiotic was included as inoculum control. After overnight incubation, colony counting of each treatment was performed to analyze the generation of resistant population. The mutant prevention concentration (MPC) was defined as the lowest concentration of antibiotic yielding no growth of resistant mutants. Selected mutants were checked for susceptibility profiles by using a Sensititre custom plate (Thermo Fisher Scientific, Massachusetts, USA) for Gram-negative bacteria, following the manufacturer’s instructions. The MICs of tetracycline, tigecycline, and doxycycline were determined using E-test diffusion strips (Biomerieux, Marcy-l'Étoile, France) on MHA.

### Whole-genome sequencing and variant calling

Up to four minocyclines and four minocycline plus RW01 resistant mutants, obtained from each of the three highest antibiotic concentration yielding resistant mutants were characterized through whole-genome sequencing (WGS). Genomic DNA of the derived one-step mutants was extracted using the High Pure PCR Template Preparation Kit (Roche Diagnostics, Basel, Switzerland). Indexed paired-end libraries were generated by using the commercial Illumina DNA Prep library preparation kit (Illumina Inc., San Diego, CA, USA). All samples were then sequenced using a MiSeq desktop sequencer cartridge (MiSeq Reagent Kit, version 3; Illumina). The reads for each isolate were mapped against the genome of the *P. aeruginosa* reference strain PAO1 (RefSeq accession number NC_002516.2) using Bowtie 2 software, version 2.2.6 (http://bowtie-bio.sourceforge.net/bowtie2/index.shtml) (29). Pileups and raw files of the mapped reads were obtained by using SAMtools, version 0.1.16 (https://sourceforge.net/projects/samtools/files/samtools/) (30), and PicardTools, version 1.140 (https://github.com/broadinstitute/picard). For variant identification, read alignments surrounding all putative insertions and deletions (Indels) were realigned using the Genome Analysis Toolkit, version 3.4–46 (https://www.broadinstitute.org/gatk/) (31). From the raw files, single-nucleotide polymorphisms (SNPs) were listed when meeting the following criteria: a quality score of >50, a root mean square (RMS) mapping quality of >25, and a coverage depth of >30. Indels were extracted from the total pileup files by using the following criteria: a quality score of >250, an RMS mapping quality of >25 and a coverage depth of >30 (32). SNPs and Indels for each isolate were annotated by using SnpEff software, version 4.3 (http://snpeff.sourceforge.net/index.html) (33), with default options. SNP and Indel compilations from the parental strains were used to filter the initial SNPs/Indels present in the parental PAO1 strain prior to the initiation of the antibiotic exposure experiments. Finally, large chromosomal deletions were analyzed with SeqMonk, version 1.47.2. (https://www.bioinformatics.babraham.ac.uk/projects/seqmonk/).

### Permeability assays

To assess the ability of RW01 to permeabilize the bacterial membrane, visualization on high-resolution microscopy and flow cytometry assays were performed. The BacLight LIVE/DEAD Bacterial Viability and Counting Kit (Thermo Fisher Scientific, Ref. L34856) was used. The kit contains SYTO9 stain for live cells with intact membranes, emitting green fluorescence (excitation/emission wavelength = 485/498 nm), and propidium iodide (PI) that penetrates and stains cells with compromised membranes, emitting red fluorescence (excitation/emission wavelength = 535/617 nm). PAO1 strain liquid culture was prepared according to the manufacturer’s instructions. After washing with NaCl 0.9%, PAO1 suspension was incubated for 4 and 24 h with 0.2× MIC, 0.5× MIC, 1.0× MIC, 5.0× MIC, and 10.0× MIC, as well as with colistin (control treatment) at the same MIC proportions, where 1 mg/L is the MIC for colistin. After both incubation times, cells were washed with 0.9% NaCl and stained with 1.5 µL of 1:1 ration mix of SYTO9 and PI. The mixture was incubated in the dark at room temperature for 15 min.

With a final volume of 50 µL, permeable and non-permeable bacterial populations were differentiated based on their fluorescence emission profiles, and the proportion of SYTO9-stained cells vs PI-stained cells was calculated from the total number of events recorded. MACSQuant Analyzer 10 Cytometer was used for this experiment, and MACSQuantify Software was used for results analysis. A two-way analysis of variance (ANOVA) with multiple comparisons was conducted to assess the effects of treatment (RW01 vs colistin), concentration, and time (4 h vs 24 h) on permeabilization and PI^+^ acquisition. A *P* value of <0.05 was considered for statistical significance. For microscopy visualization, an inverted confocal microscope was used (Leica Microsystems, Wetzlar, Germany).

### Uptake of minocycline

To check whether RW01 leads to a major intracellular minocycline accumulation, the following protocol was adapted from previous studies (15, 34). A liquid culture of wild-type PAO1 strain was incubated overnight at 37°C, 180 rpm. Then, OD_600_ was adjusted to 0.05 and incubated again to mid-log phase culture. PAO1 was then washed and resuspended in 10 mM of commercial HEPES buffer at ⁓10^8^ CFU/mL. A fresh stock of minocycline was prepared at 2 mg/mL and further diluted in HEPES buffer. In a Corning 96-well black plate with clear bottom, direct bacterial culture and minocycline were added to a final concentration of 128 mg/L, reaching a final volume of 190 µL. Fluorescence of minocycline was measured in a TECAN Infinite M Nano+ 200 Pro microplate reader, with 405 nm excitation wavelength and 535 nm emission wavelength for 6 min every 2 min. Immediately after, 10 µL of RW01 was added to a final concentration of 1× MIC (500 mg/L) and 0.2× MIC (100 mg/mL) in three different replicate wells. As permeabilization treatment control, 10 µL colistin was used to a final concentration of 1 mg/L (1× MIC) and 30 mg/L, again in triplicate. A negative control was also added, substituting RW01/colistin by MilliQ water. Fluorescence was measured continuously for 2 h, and the results were expressed as fluorescence readings, calculated by subtracting the background fluorescence of the untreated bacterial culture from each sample. Three independent replicates were performed, and mean data were plotted. A two-way ANOVA test was applied for statistical analysis.

### *In vivo* acute and cumulative toxicity

To study the acute toxicity of RW01, two groups of *n* = 6 C57BL/6J females (≈20 g) were treated intraperitoneally (ip) with a single dose of 0.2 mL of 250 and 500 mg/L concentrations of RW01, respectively. After administration and for 7 days, the following indicative signs of pain were assessed: reduced water (dehydration), food intake, isolation, self-mutilation, tremors/spasms, dyspnea, physical activity (increased/reduced), chromodacryorrhea, muscle stiffness, piloerection, teeth grinding, and weight loss. For cumulative toxicity determination, a group of *n* = 6, 7-week-old healthy C57BL/6J females was treated ip, with a 0.2 mL single dose per day for 3 days at a concentration equivalent to 500 mg/L. After administration and for 7 days, the same indicative signs of pain/toxicity detailed in the acute toxicity studies were evaluated.

## RESULTS

### Antimicrobial susceptibility testing and synergy assessment

**Fig 2:**
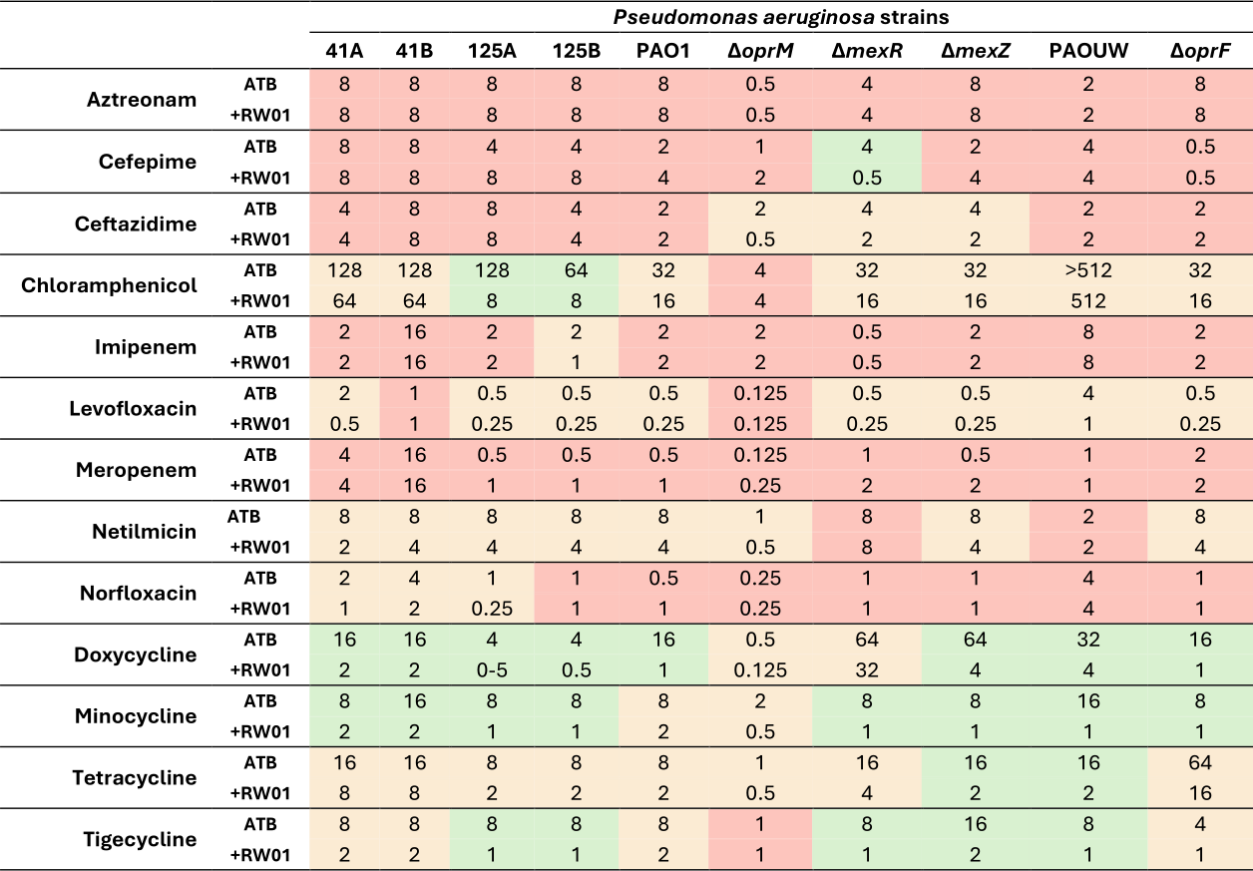
Minimum inhibitory concentration of each antibiotic with and without 100 mg/L of RW01. In green, ≥4-fold MIC reduction; in orange, 1- to 4-fold MIC reduction; in red, ≤1-fold MIC reduction.

**Fig 3:**
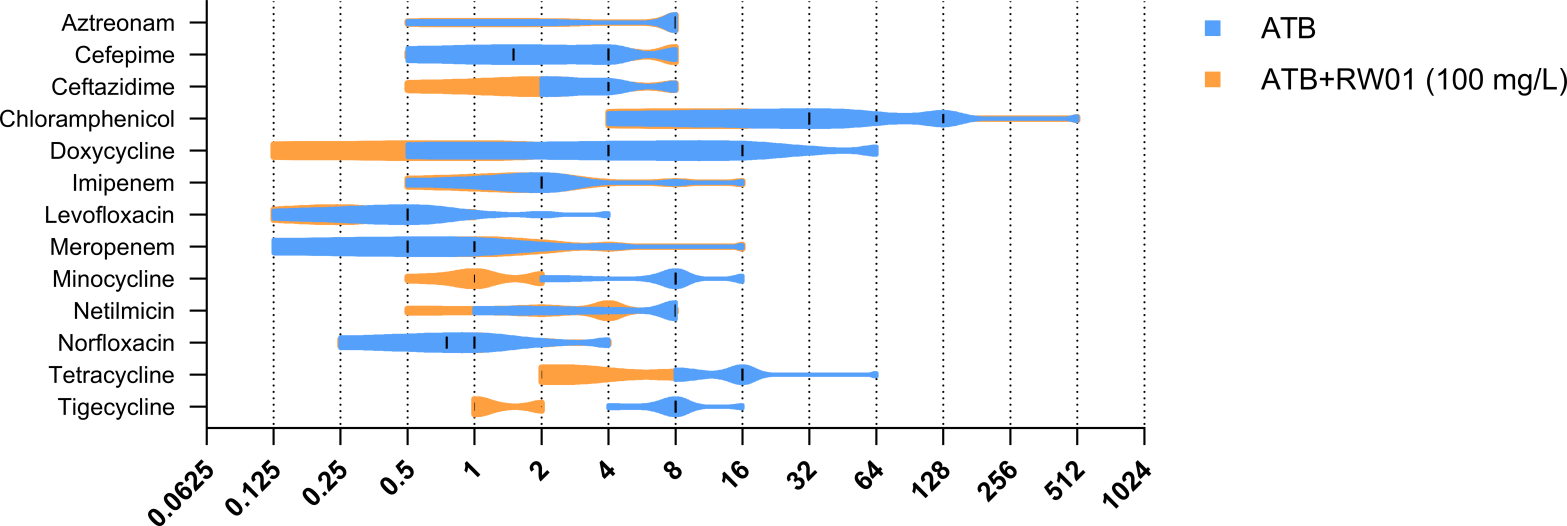
MIC distribution of antibiotic vs combined treatment with RW01 among the *P. aeruginosa* strains used in this study. The *x* axis represents the concentration of the antibiotic used in milligram per liter. The *y* axis represents all the antibiotics tested. In blue, MIC distribution of antibiotic itself; in orange, MIC distribution of the combination. ATB, antibiotic.

After establishing the susceptibility profiles of RW01, a pattern of activity in combination with tetracyclines was observed. The complete MIC values for each antibiotic, both in the presence and absence of 100 mg/L of RW01, are detailed in Fig. 2. Notably, RW01 demonstrated a significant potentiation effect, evidenced by an ≥8-fold reduction in the MIC of specific isolates such as *P. aeruginosa* strains 125A and 125B when incubated with chloramphenicol and of the PAO1Δ*mexR* strain when incubated with cefepime, where the MIC decreased from 4 to 0.5 mg/L, respectively, upon combination with RW01. However, a broader-spectrum enhancement of activity was observed involving tetracycline-class antibiotics, including tetracycline, minocycline, tigecycline, and doxycycline. Doxycycline exhibited up to a 16-fold MIC reduction, with MIC values decreasing from 16 mg/L (doxycycline alone) to 1 mg/L when combined with RW01 in PAO1. Similarly, minocycline showed a significant eightfold reduction, with MICs decreasing from 16 and 8 mg/L to 1–2 mg/L (strains 41B and PAOUW) and 1 mg/L (strains 125A, 125B, PAOΔ*mexR,* PAOΔ*mexZ*, and PAOUWΔ*oprF*), respectively. Similar results were observed for tigecycline, where all strains exhibited four- to eightfold reductions in MIC. In contrast, tetracycline displayed the weakest interaction with RW01 among the antibiotics in its class, showing maximum MIC reductions of fourfold magnitude. The distribution of MIC values for each antibiotic, both alone and in combination with RW01, is presented in Fig. 3. The figure illustrates that, generally, a minimum of two- to eightfold reduction in MIC was consistently observed across the tested population when tetracyclines were co-administered with RW01.

**Fig 4:**
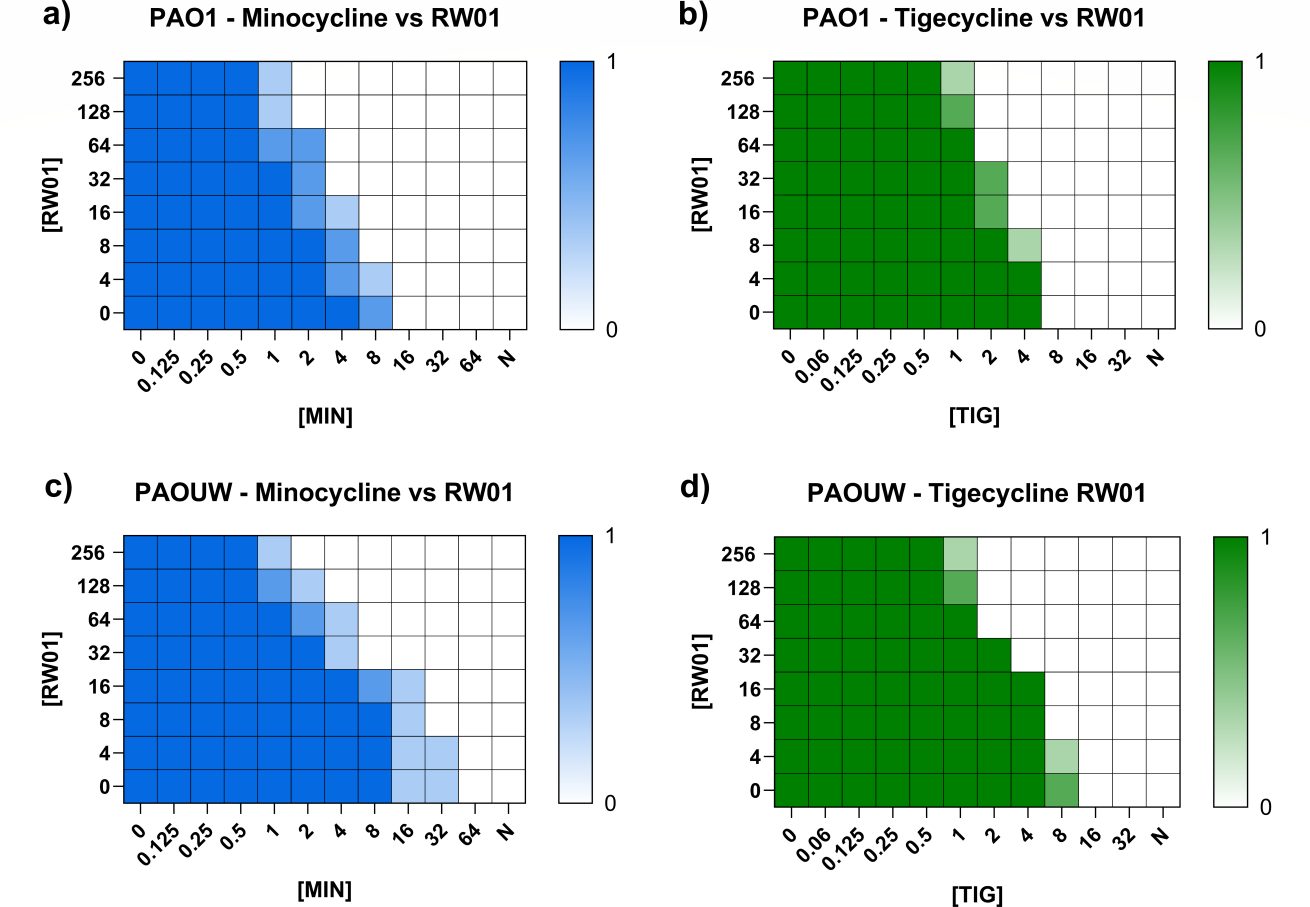
Results of checkerboard assays performed with PAO1 (a and b) and PAOUW (c and d). Color intensity indicates the frequency of observed growth: 0 (white) represents no growth, while 1 (dark blue/green) indicates growth observed in all replicates. Light blue/green denotes growth observed in one replicate; medium blue/green denotes growth observed in two replicates. On the *x* axis, minocycline and tigecycline concentration in milligram per liter. On the *y* axis, RW01 concentration in milligram per liter. N, negative control column.

Checkerboard assays combining minocycline and tigecycline with RW01 in the wild-type reference strains yielded average FIC Index values of 0.535 and 0.677 for PAO1, respectively, and 0.406 and 0.446 for PAOUW (Fig. 4). These results suggest an additive interaction between RW01 and both tetracyclines in PAO1 and synergistic interaction in PAOUW. In addition to conducting checkerboard assays, FICI values were calculated to assess whether 100 mg/L of RW01 exerts a synergistic effect on the activity of tetracyclines (Table 2). In the majority of cases, the combination of 100 mg/L RW01 with tetracyclines demonstrated a synergistic interaction, as they all showed FICI values lower than 0.5. Exceptionally, the combination of RW01 with doxycycline in the PAO1Δ*mexR* strain resulted in an additive effect, with a FICI of 0.598. Similarly, additive effects were observed in strains 41A, 41B, and PAO1Δ*oprM* when combined with tetracycline, yielding an FICI value of 0.598 in all instances. Notably, there was a single case in which RW01 exhibited an indifferent interaction, which occurred with tigecycline in the PAO1Δ*oprM* strain, where the FICI was 1.098.

**TABLE 2:** Synergy assessment of RW01 at 100 mg/L and doxycycline/minocycline/tetracycline/tigecycline for each *P. aeruginosa* strain

Strain	RW01 MIC	Antibiotic MIC	Antibiotic MIC + RW01 100 mg/L	Potentiation (fold)	FICI^*a*^	FICI-determined effect
Doxycycline						
*P. aeruginosa* 41A	1,024	16	2	8	0.223	Synergy
*P. aeruginosa* 41B	1,024	16	2	8	0.223	Synergy
*P. aeruginosa* 125A	512	4	0.5	8	0.320	Synergy
*P. aeruginosa* 125B	512	4	0.5	8	0.320	Synergy
*P. aeruginosa* PAO1	512	16	1	16	0.258	Synergy
*P. aeruginosa* PAOΔ*oprM*	1,024	0.5	0.125	4	0.348	Synergy
*P. aeruginosa* PAOΔ*mexR*	1,024	64	32	2	0.598	Additivity
*P. aeruginosa* PAOΔ*mexZ*	1,024	64	4	16	0.160	Synergy
*P. aeruginosa* PAOUW	1,024	32	4	8	0.223	Synergy
*P. aeruginosa* PAOΔ*oprF*	1,024	16	1	16	0.160	Synergy
Minocycline						
*P. aeruginosa* 41A	1,024	8	2	4	0.348	Synergy
*P. aeruginosa* 41B	1,024	16	2	8	0.223	Synergy
*P. aeruginosa* 125A	512	8	1	8	0.320	Synergy
*P. aeruginosa* 125B	512	8	1	8	0.320	Synergy
*P. aeruginosa* PAO1	512	8	2	4	0.445	Synergy
*P. aeruginosa* PAOΔ*oprM*	1,024	2	0.5	4	0.348	Synergy
*P. aeruginosa* PAOΔ*mexR*	1,024	8	1	8	0.223	Synergy
*P. aeruginosa* PAOΔ*mexZ*	1,024	16	1	16	0.160	Synergy
*P. aeruginosa* PAOUW	1,024	8	1	8	0.223	Synergy
*P*. aeruginosa PAOΔ*oprF*	1,024	8	1	8	0.223	Synergy
Tetracycline						
*P. aeruginosa* 41A	1,024	16	8	2	0.598	Additivity
*P. aeruginosa* 41B	1,024	16	8	2	0.598	Additivity
*P. aeruginosa* 125A	512	8	2	4	0.445	Synergy
*P. aeruginosa* 125B	512	8	2	4	0.445	Synergy
*P. aeruginosa* PAO1	512	8	2	4	0.445	Synergy
*P. aeruginosa* PAOΔ*oprM*	1,024	1	0.5	2	0.598	Additivity
*P. aeruginosa* PAOΔ*mexR*	1,024	16	4	4	0.348	Synergy
*P. aeruginosa* PAOΔ*mexZ*	1,024	16	2	8	0.223	Synergy
*P. aeruginosa* PAOUW	1,024	64	16	4	0.348	Synergy
*P. aeruginosa* PAOΔ*oprF*	1,024	16	2	8	0.223	Synergy
Tigecycline						
*P. aeruginosa* 41A	1,024	8	2	4	0.348	Synergy
*P. aeruginosa* 41B	1,024	8	2	4	0.348	Synergy
*P. aeruginosa* 125A	512	8	1	8	0.320	Synergy
*P. aeruginosa* 125B	512	8	1	8	0.320	Synergy
*P. aeruginosa* PAO1	512	8	2	4	0.445	Synergy
*P. aeruginosa* PAOΔ*oprM*	1,024	1	1	1	1.098	Indifference
*P. aeruginosa* PAOΔ*mexR*	1,024	8	1	8	0.223	Synergy
*P. aeruginosa* PAOΔ*mexZ*	1,024	8	1	8	0.223	Synergy
*P. aeruginosa* PAOUW	1,024	4	1	4	0.348	Synergy
*P. aeruginosa* PAOΔ*oprF*	1,024	16	2	8	0.223	Synergy

^*a*^ Fractional Inhibitory concentration index was calculated as follows: FICI = (MIC_RW01 100 + ATB_ / MIC_RW01_) + (MIC_RW01 100 + ATB_ / MIC_ATB_).

### Resistance mechanism characterization

**Fig 5:**
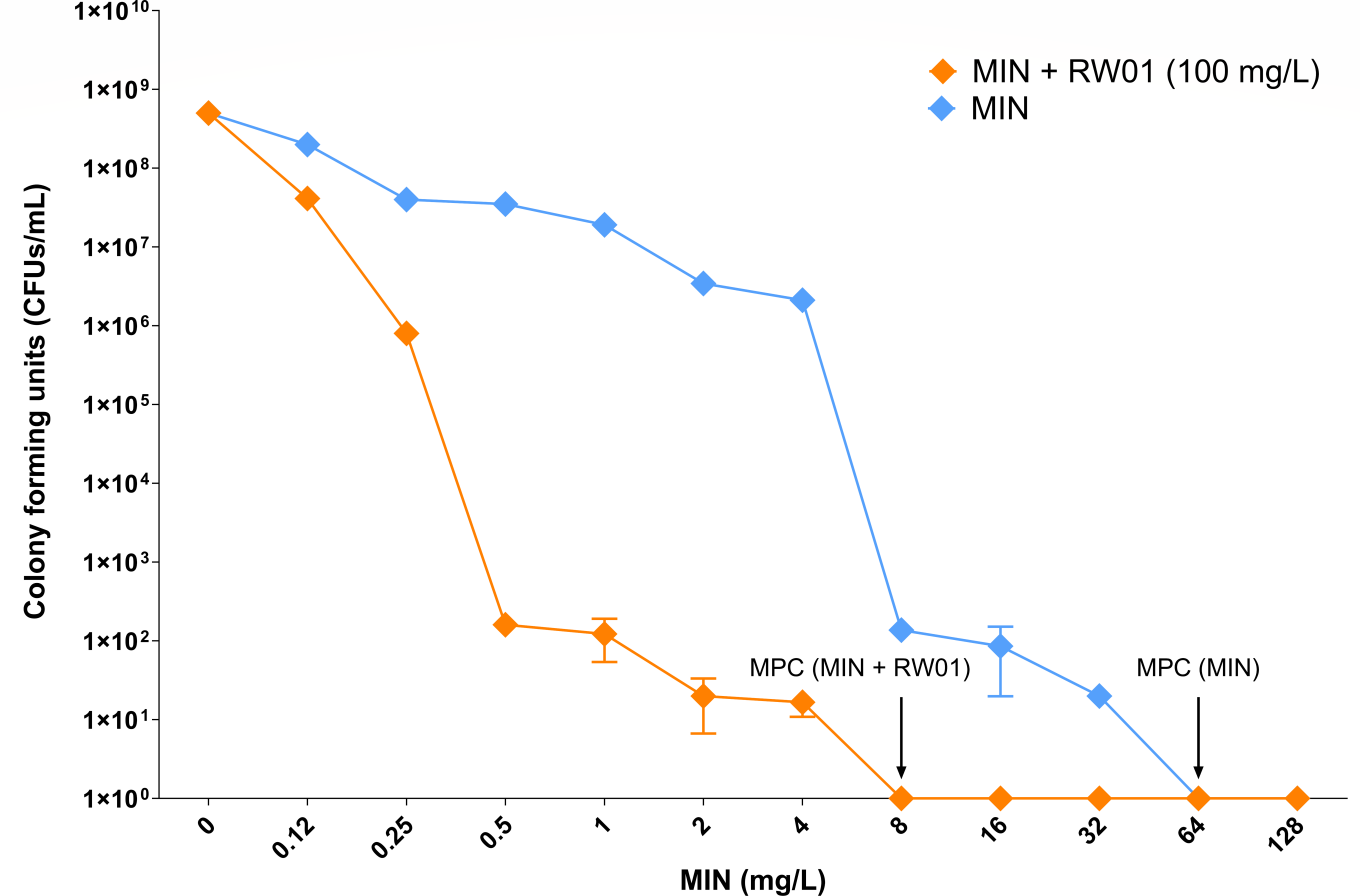
Population analysis through CFU per milliliter recounts of PAO1 in increasing concentrations of minocycline alone or in combination with RW01 at 100 µg/mL. MPC, mutant prevention concentration.

Population studies illustrated in Fig. 5 revealed MICs of minocycline for wild-type *P. aeruginosa* PAO1 ranging from 4 to 8 mg/L. However, when combined with 100 mg/L of RW01, the MICs were markedly reduced, ranging from 0.5 to 1.0 mg/L. Furthermore, the MPC demonstrated a significant reduction with the combination treatment, decreasing from 32–64 mg/L for minocycline alone to 8 mg/L when co-administered with RW01.

Analysis of the resistance mechanisms in the spontaneous mutants exhibiting the highest levels of resistance revealed that, as anticipated, resistance to minocycline was primarily driven by mutations in genes encoding repressor proteins such as NalD, NalC, MexR, or NfxB, resulting in the overproduction of the MexAB-OprM and MexCD-OprJ efflux pumps, respectively. Additionally, mutations in genes related to the synthesis of type IV pili were detected in most mutants, suggesting also a potential role in minocycline resistance. In contrast, mutants generated under the combination of minocycline and RW01 predominantly exhibited mutations in the *pmrB* gene, which is associated with LPS modification and cross-resistance to colistin (Table 3). Upon examining the MICs of RW01 for the tested mutants, no significant reduction was observed when RW01 was used alone, with MIC values remaining consistent in the range of 512–1024 mg/L. However, the MICs for the minocycline-RW01 combination exhibited notable increases, rising from 0.5–1.0 mg/L to 4.0–8.0 mg/L and reaching as high as 16 mg/L in the case of the mutant MIN4_RW01_3. For spontaneous minocycline-resistant mutants, the MICs for the combination treatment showed smaller changes compared to those observed in mutants selected by the combination treatment, with only a twofold increase in MIC relative to the wild type and, in one case, a fourfold increase in the MIC of mutant MIN16_3. The MICs for minocycline alone increased similarly across both mutant types, with values reaching up to 64 mg/L, though they typically remained within the range of 16–32 mg/L. In addition, as expected, cross-resistance to all other tetracyclines was observed in mutants from both groups. Susceptibility assays performed with Sensititre custom plates confirmed the presence of cross-resistance to colistin in mutants derived from the combination treatment, with colistin MICs increasing from 1 to 2–4 mg/L in most cases and reaching 16 mg/L in the mutant MIN4_RW01_4. This cross-resistance was not observed in mutants generated by minocycline alone, where colistin MICs remained unchanged, strengthening the idea of an interaction with bacterial LPS.

**TABLE 3:** Phenotypic and genotypic characterization of PAO1 reference strain and its derivative one-step mutants^*a*^*^,^*^*b*^*^,^*^*c*^

Strain	Genotype	MHB microdilution				Sensititre												E-test		
		RW01	MIN	MIN + RW01	TIC	*P*/TZ	AZT	TAZ	C/T	CZA	FEP	AMI	TOB	IMI	MER	COL	CIP	TET	TGC	DOX
					*R* > 16	*R* > 16	*R* > 16	*R* > 8	*R* > 4	*R* > 8	*R* > 8	*R* > 16	*R* > 2	*R* > 4	*R* > 8	*R* > 4	*R* > 0.5			
PAO1	WT	512	4–8	0.5–1	16	≤4	2–4	1–2	≤0.5/4.0	1–2	≤1	≤2	0.25–0.5	1–2	1–2	1	≤0.12	24	6–8	32
MIN1_RW01_1	*pmrB*(Y98N)	1,024	16	4	16	≤4	≤2	≤1	≤0.5	1	≤1	4	1	2	1	2–4	≤0.12	192	24	96
MIN1_RW01_2	*mexR*(G58E), *pilB*(G326D), *hutC*(Y181X)	1,024	32–64	2–4	128	16	16	4	≤0.5	4	4	≤2	≤0.25	1	4	1	0.5	256	32	256
MIN1_RW01_3	*pmrB*(V215L)	1,024	16	4	16	≤4	≤2	≤1	≤0.5	1	≤1	4	1	2	1	8	0.25	256	16	64
MIN1_RW01_4	*pmrB*(F124L)	1,024	32	4	16	8	8	2	≤0.5	2	2	≤2	≤0.25	2	1	2	0.25	64	24	96
MIN2_RW01_1	*pmrB*(V215L)	2,048	32	8–16	64	≤4	4–8	2	≤0.5	1	2	4	0.5	4	1	4	0.25	128	24	96
MIN2_RW01_2	*pmrB*(M292T)	1,024	16–32	4	32	8	4	2	0.5–1.0	2	4	4	0.5	2	1	2	0.25–0.5	192	16	128
MIN2_RW01_3	*parS*(V152A)	1,024	16	0.5	16	4–8	4	2	1	1	4	8	1	8	2–4	1	0.5	256	48	256
MIN2_RW01_4	*pmrB*(M292T)	2,048	32	4	32	8	8	2	≤0.5	2	4	4	0.5	2	1	4	0.5–1	96	16	64
MIN4_RW01_1	*pmrB*(R429H)	1,024	32	8	128	16	16	4	≤0.5	4	4–8	2–4	0.5	2–4	4	2	0.5	256	16	256
MIN4_RW01_2	*pmrB*(F124S)	2,048	32	4	16	≤4	≤2	≤1	≤0.5	1	≤1	4	0.5	2	≤0.5	4	≤0.12	64	16	64
MIN4_RW01_3	*pmrB*(M292T)	2,048	64	16	16	≤4	≤2	≤1	≤0.5	1	≤1	4	0.5	16	2	4	0.25	256	32	128
MIN4_RW01_4	*pmrB*(Y98N)	2,048	32	8	16	≤4	≤2	≤1	≤0.5	≤0.5	≤1	4	1	4	1	16	0.25	96	32	64
MIN8_1	*nalD*(R3C), *pilM*(Q62X)	512	16	2	64	8	8	4	≤0.5	4	4	≤2	0.5	2	2	1	0.25	256	32	256
MIN8_2	*pilZ*(nt18InsT)	1,024	16	2	64	8	8	4	≤0.5	2–4	4	≤2	0.5	2	2	1	0.25	256	32	256
MIN8_3	*pilZ*(nt18InsT)	1,024	16	2	64	8	16	4	1	4	4	≤2	≤0.25	2	2	1	0.5	64	16	128
MIN8_4	*pilZ*(nt18InsT)	1,024	16	2	64	16	16	4	1	4	4	≤2	0.5	2	2	1	0.5	64	32	256
MIN16_1	*nalD*(T43P)	512	16	2	64	8	16	4	≤0.5	4	4	4	0.5	2–4	4	1	0.25	256	16	256
MIN16_2	*nalC*(aa9∆4), *pilQ*(nt46∆1)	512	16	2	64	8–16	8–16	4	≤0.5	4	4	≤2	≤0.25	2	2	2	0.25	96	32	128
MIN16_3	*nfxB*(nt79∆14), *pilB*(N349K)	512	16–32	4	16	≤4	2–4	2	≤0.5	1	4	≤2	≤0.25	1	≤0.5	1	4	128	48	192
MIN16_4	*nalC*(T50P), *pilM*(Q62X)	1,024	16	2	64	8	16	4	0.5–1.0	2–4	4	≤2	≤0.25	2	2	1	0.5	64	16	128
MIN32_1	*mexR*(R63H), *pilB*(N349K), PA0646(V316G)	1,024	32–64	2	128	16	16	4	≤0.5	4	4	≤2	0.25–0.5	2	4	2	0.5	256	24	256

^*a*^ The name of each mutant corresponds to the concentration of minocycline they were isolated and also if RW01 was present or not.

^*b*^ AMI, amikacin; AZT, aztreonam; CIP, ciprofloxacin; COL, colistin; C/T, ceftolozane/tazobactam 4; CZA, ceftazidime/avibactam; DOX, doxycycline; FEP, cefepime; IMI, imipenem; MER, meropenem; MIN, minocycline; ND, not determined; P/TZ, piperacillin/tazobactam; TAZ, ceftazidime; TET, tetracycline; TGC, tigecycline; TIC, ticarcillin; TOB, tobramycin.

^*c*^ EUCAST breakpoint v.14.0 was used.

### Permeabilizing activity of RW01 and minocycline uptake

**Fig 6:**
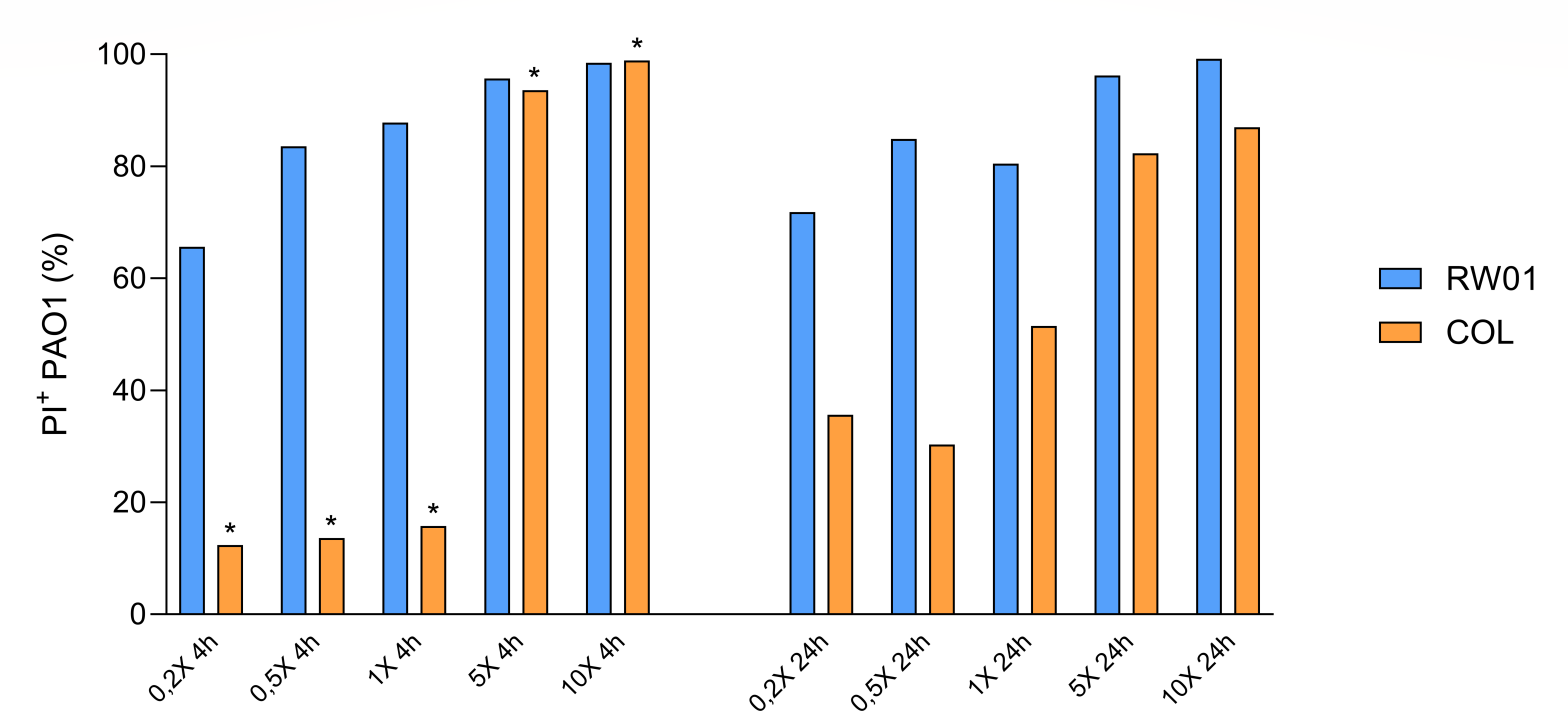
PI^+^ percentage (%) obtained from flow cytometry recounts of wild-type PAO1, at different concentrations of RW01 (0.2× MIC, 100 mg/L; 0.5× MIC, 250 mg/L; 1× MIC, 500 mg/L; 5× MIC, 2.5 g/L; and 10× MIC, 5 g/L) and colistin (0.2× MIC, 0.2 mg/L; 0.5× MIC, 0.5 mg/L; 1× MIC, 1 mg/L; 5× MIC, 5 mg/L; and 10× MIC – 10 mg/L) and at different times (4 and 24 h). *Difference between 4 h RW01 treatment and 4 h colistin treatment was statistically significant.

**Fig 7:**
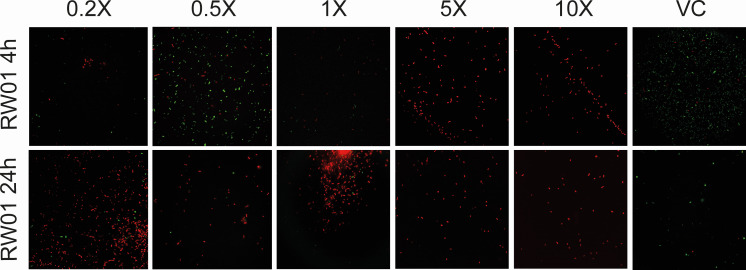
Visualization of RW01’s permeabilization activity in PAO1 at increasing concentrations under confocal microscopy (0.2× MIC, 100 mg/L; 0.5× MIC, 250 mg/L; 1× MIC, 500 mg/L; 5× MIC, 2.5 g/L; and 10× MIC, 5 g/L). VC, viability control, no treatment.

Flow cytometry revealed that the 65.6% of the counted bacteria were permeabilized leading to PI accumulation at 4 h when treated with 0.2× MIC RW01 (100 mg/L), in contrast to the 12.4% PI^+^ of colistin 0.2× MIC (0.2 mg/L) at 4 h. Percentage of permeabilization increases evenly as RW01 concentration also increases. Contrarily to colistin treatment, that abruptly increases PI^+^ recounts when switching concentrations to 1× MIC to 5× MIC, from 15.8% to 93.6%, respectively. At 24 h recount, a maximum of 71.8% of PI^+^ bacteria were detected when treated with 0.2× MIC RW01, increasing up to 99.2% when treated at 10× MIC. Similar levels of permeabilization were detected at 24 h when treated with colistin at 10× MIC (Fig. 6). Tukey’s multiple comparison two-way ANOVA revealed a statistical difference between RW01 and colistin at 4 h with an adjusted *P* value = 0.0168 (mean difference (diff.) 3,938; 95% confidence interval [CI] 6,832–71.93), contrary to treatments at 24 h, with a non-significant *P* value of 0.0844 (mean diff. 2,918; 95% CI 3,368–61.73). Additionally, there was no significant difference between treatment times (4 and 24 h) in RW01, remarking the rapid action of RW01 to permeabilize the bacterial membrane. Complete cytometry plots can be found in detail at the Supplemental Material section. Confocal visualization of PAO1 treated with RW01 is represented in Fig. 7.

**Fig 8:**
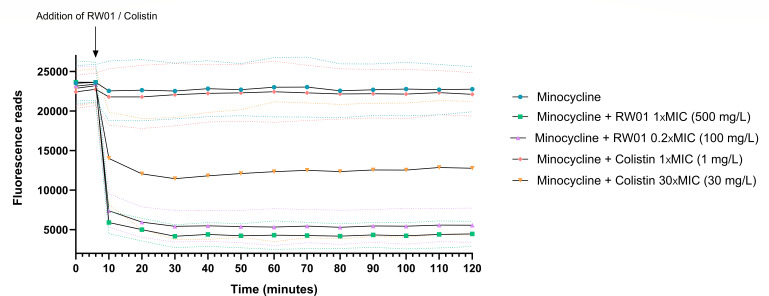
Fluorescence peak measurements of minocycline that relate to intracellular accumulation. Dotted lines represent error bars of the three replicates for each treatment.

To determine whether this demonstrated permeabilization boosts minocycline accumulation within the bacterial cell, minocycline uptake was monitored in the presence of RW01, with colistin serving as a control. Figure 8 shows a peak in fluorescence detection of minocycline when RW01 was added at 100 and 500 mg/L (0.2× MIC and 1.0× MIC, respectively) compared to control detection of minocycline in PAO1 in which only water was added at 6 min. Control treatment with colistin showed less decrease in fluorescence detection. Statistical analysis determined that both treatments with RW01 were significantly different with the no-treatment control, with adjusted *P* values of <0.0001 (mean diff. 17,128 [95% CI 15,945–18,310] for RW01 1× MIC; mean diff. 16,082 [95% CI 14,900–17,265] for RW01 0.2xMIC). Also, statistical difference was found between RW01 1× MIC treatment and colistin 1× MIC treatment (*P* value <0.0001; mean diff. −15,508 [95% CI −16,690 to −14,326]).

### Activity loss of synthetic analogs

To evaluate the mode of action of RW01, RW01 derivatives were synthesized, and activity was tested. The results demonstrated a complete loss of the potentiating activity exhibited by the original RW01 cyclic peptide in all three analogs. No fold reduction of the MIC was observed in the PAO1 strain when comparing the antibiotic vs the combination of RW01 and antibiotic. This enlightens that the modifications introduced in these derivatives likely targeted the specific locus of the peptide responsible for its activity.

### *In vivo* toxicity and serum effect on RW01 activity

None of the doses tested of RW01 showed any of the 11 symptoms indicative of pain/toxicity during the 7 day study period in either the acute or cumulative toxicity studies. After assessing the impact of 25% mouse serum on the antimicrobial activity of RW01 (100 mg/L), the following results were obtained. The MIC of doxycycline for the PAO1 strain was 16 mg/L when tested alone and reduced to 1 mg/L in combination with RW01. These MIC values remained unchanged in the presence of mouse serum. For minocycline, the MIC values ranged from 8 to 16 mg/L when tested alone and increased to 32 mg/L in the presence of serum. In combination with RW01, the minocycline MIC increased from 2 to 4 mg/L in serum, reflecting a non-significant onefold increase. These findings suggest that the serum does not significantly alter the activity of the RW01 maintaining its activity.

## DISCUSSION

Decreased outer membrane permeability, along with the expression of efflux pumps like MexAB-OprM, is one of the main intrinsic resistance mechanisms in *P. aeruginosa*. This acts as a selective barrier to the uptake of numerous antibiotics, mainly due to the restricted size and limited number of its primary non-specific porin, OprF, which predominantly features very small channels (35). Consequently, most antibiotics used to treat *P. aeruginosa* infections must effectively penetrate the cell membrane to reach and act on intracellular targets (36, 37). In other instances, the antibiotic directly targets the cell membrane, where its activity is focused. That is the case of the polypeptides polymyxin B and colistin, which interact with the lipid A portion of the outer membrane’s LPS in Gram-negative bacteria, leading to an increase in membrane permeability and antibiotic uptake (38). The concept behind membrane-disrupting adjuvants is to permeabilize the bacterial outer membrane, similar to the action of polymyxins, thereby facilitating the entry of antibiotics. This strategy seeks to improve antimicrobial efficacy while potentially mitigating the high toxicity commonly associated with these types of agents.

To date, only direct resistance breakers, such as β-lactamase inhibitors classified as class I adjuvants, have been approved for commercialization and use in combination therapy. However, promising membrane-targeting molecules appear to be investigated (16). In 2020, the potent broad-spectrum adjuvant SLAP-S25 was reported as a membrane damage inducer by binding to LPS in the outer membrane and phosphatidylglycerol in the cytoplasmic membrane, thereby enhancing colistin efficacy in MDR Gram-negative pathogens (39). Another example of a published membrane-disrupting molecule is P35, a less toxic analog of pentamidine that significantly potentiates novobiocin (40). The main challenge for outer membrane-disrupting molecules, such as colistin, is their associated toxicity, which poses a significant barrier to the advancement of new compounds into clinical trials. In this context, the selection of RW01 for this study as antibiotic adjuvant was primarily driven by its low antimicrobial activity and toxicity, making it a promising candidate for further investigation.

Therefore, non-toxic permeabilizers, often cationic and amphiphilic or chelating agents, represent a strategy to enhance antibiotic efficacy that is constrained by the bacterial outer membrane. These compounds, which may be derived from peptides, peptide-like structures, polymers, or lipids, including antimicrobial peptides, have shown potential in this capacity (22, 41, 42). RW01 compound, as a cyclic peptide composed of proline, arginine, and tryptophan amino acids, has potential amphiphilic properties due to the hydrophobic nature of proline and tryptophan’s indole ring, combined with the hydrophilic properties of arginine’s charged side chain and tryptophan’s amine group. This structure potentially enables the peptide to interact with both hydrophobic and hydrophilic environments, presumably allowing it to interact with bacterial membranes. The results obtained with RW01 analogs showed full activity loss, arguably pointing to key polar interactions of the amino group in the D-Pro(NH_2_) residue that the corresponding amide analogs cannot emulate. Future studies will focus on analogs that preserve a free amino group on D-Pro(NH2) to further evaluate the mode of action.

Whole-genome sequencing of resistant mutants confirmed that resistance to RW01/minocycline combination is somehow modulating the composition of the outer membrane. LPS modification is known to be regulated by the two-component systems PhoPQ and PmrAB in response to limiting magnesium concentrations (43, 44). Modification of lipid A by the addition of phosphoethanolamine has been demonstrated to be linked to mutations in the *pmrA*/*pmrB* genes, resulting in resistance to colistin by preventing its interaction with LPS (45). The observation that nearly all RW01/minocycline-resistant mutants harbor mutations in *pmrB* suggests the involvement of LPS modification. This is further corroborated by the observed cross-resistance to colistin specifically in the *pmrB* mutants. On the other side, as expected, minocycline-resistant mutants predominantly exhibited mutations in genes that regulate efflux pumps responsible for the expulsion of tetracyclines (46, 47). Although addressing cross-resistance to colistin may become a future challenge, it is reassuring that the combination therapy of RW01 and minocycline significantly reduces the MPC compared to minocycline monotherapy. This reduction is potentially due to the minor selective pressure exerted by RW01 on PAO1, driven by its low antimicrobial activity. However, despite these insights, the precise mechanism by which RW01 enhances permeability and facilitates the selective entry of tetracyclines remains unclear, and further exploration of RW01’s structural characteristics is expected to shed light on this.

The potential permeabilizing activity of RW01 on bacterial membranes was further evaluated using flow cytometry, spectrophotometry, and microscopy. Propidium iodide can only enter cells when the bacterial membrane is compromised or the cells are dead, while SYTO-9 can penetrate healthy bacteria (48). Flow cytometry recounts indicated that, after 24 h, over 70% and 80% of the bacteria treated with RW01 at 0.2 and 0.5 of the growth-inhibitory concentration, respectively, exhibited permeability changes and were detected as PI^+^. This suggests that RW01 enhances membrane permeability without necessarily inducing cell death. Furthermore, fluorescence analysis of minocycline revealed a distinct emission pattern, indicating that RW01 significantly impacts minocycline accumulation. This observation suggests that RW01’s permeabilizing effect may facilitate the entry of minocycline into the intracellular space.

The primary therapeutic options for treating infections caused by *P. aeruginosa* include fluoroquinolones, β-lactams plus β-lactamase-inhibitors, and aminoglycosides (47). In this species, the effectiveness of tetracyclines is constrained by active efflux mechanisms and low membrane permeability, even for eravacycline, a newer tigecycline derivative with a broader spectrum of activity against Gram-negative bacteria, specifically designed to overcome resistance to conventional antitetracycline mechanisms (9, 49, 50). Nevertheless, the synergistic effect of RW01 on all tested tetracyclines suggests that these antimicrobials may regain efficacy, as RW01 has been shown to lower the MIC below susceptibility/intermediate breakpoints established in other species in many instances. This includes the EUCAST tigecycline susceptibility breakpoint of ≤2 mg/L for Enterobacteriaceae, the minocycline and tetracycline susceptibility breakpoints of ≤1 or ≤4 mg/L for methicillin-resistant *Staphylococcus aureus* (MRSA) according to EUCAST or CLSI, respectively, and the EUCAST doxycycline intermediate breakpoint of 0.5 mg/L for *Streptococcus pneumoniae* (51–53). Surprisingly, no additional significant activity was observed with any of the other antimicrobials tested, but the underlying reason for RW01’s specificity towards tetracycline-like antibiotics remains unclear and requires further investigation. The mode of action could not be successfully evaluated to gather further information, as all derivatives intended for use in protein-interaction assays lost their activity, resulting in the discontinuation of this line of investigation. However, reports of synergy between colistin and either minocycline or tigecycline, often demonstrating stronger effects than those observed in this study, support the hypothesis that membrane disruptors enhance the intracellular uptake of tetracyclines (54–56).

*In vivo* studies successfully served as a preliminary step in assessing RW01’s toxicity, paving the way for the next critical step in *in vivo* assays: efficacy determination. Most peptides are known to lack certain pharmacological properties (57, 58). In this regard, RW01’s serum resistance has been confirmed, showing stable activity in serum-embedded MICs, which appear unaffected by serum proteases. However, other properties such as tissue penetration, bioavailability, and elimination still need to be evaluated as part of RW01’s activity characterization and may represent potential limitations to address. Additionally, to account for any limitations in this study, future work should incorporate a larger and more genetically and geographically diverse sample of *P. aeruginosa*, allowing for more generalizable results and enhancing the reliability of both *in vitro* and *in vivo* findings.

In conclusion, the mechanistic studies presented underscore the potential of membrane-disturbing adjuvants in mitigating antimicrobial resistance for specific antibiotics, offering them a renewed opportunity in an era where alternative strategies are critically needed. The primary objective is to minimize the common toxicity associated with membrane-targeting agents while also reducing antimicrobial activity to prevent the rapid emergence of resistance-providing mutations. Given the preliminary non-toxicity of RW01 *in vivo*, this peptide in combination therapy shows considerable promise. Further research is warranted to evaluate its *in vivo* efficacy and cytotoxicity.

## SUPPLEMENTAL MATERIAL

Supplemental material aac.01459-24-s0001.pdf Synthesis of RW01 peptide analogues, and flow cytometry analysis.

ASM does not own the copyrights to Supplemental Material that may be linked to, or accessed through, an article. The authors have granted ASM a non-exclusive, world-wide license to publish the Supplemental Material files. Please contact the corresponding author directly for reuse.

## References

[1] World Health Organization. 2024. WHO bacterial priority pathogens list, 2024. Available from: https://www.who.int/publications/i/item/WHO-EMP-IAU-2024

[2] Oliver A, Rojo-Molinero E, Arca-Suarez J, Beşli Y, Bogaerts P, Cantón R, Cimen C, Croughs PD, Denis O, Giske CG, et al.. 2024. Pseudomonas aeruginosa antimicrobial susceptibility profiles, resistance mechanisms and international clonal lineages: update from ESGARS-ESCMID/ISARPAE Group. Clin Microbiol Infect 30:469–480. doi:10.1016/j.cmi.2023.12.026 38160753

[3] López-Causapé C, Cabot G, Del Barrio-Tofiño E, Oliver A. 2018. The versatile mutational resistome of Pseudomonas aeruginosa. Front Microbiol 9:685. doi:10.3389/fmicb.2018.00685 29681898 PMC5897538

[4] World Health Organization. 2017. Guidelines for the prevention and control of carbapenem-resistant Enterobacteriaceae, Acinetobacter baumannii and Pseudomonas aeruginosa in health care facilities. Available from: http://apps.who.int/iris/bitstream/10665/259462/1/9789241550178-eng.pdf?ua=1%0Ahttp://www.who.int/infection-prevention/publications/guidelines-cre/en/29630191

[5] Antimicrobial Resistance Collaborators. 2022. Global burden of bacterial antimicrobial resistance in 2019: a systematic analysis. Lancet 399:629–655. doi:10.1016/S0140-6736(21)02724-0 35065702 PMC8841637

[6] Oliver A, Mulet X, López-Causapé C, Juan C. 2015. The increasing threat of Pseudomonas aeruginosa high-risk clones. Drug Resist Updat 21–22:41–59. doi:10.1016/j.drup.2015.08.002 26304792

[7] Du D, Wang-Kan X, Neuberger A, van Veen HW, Pos KM, Piddock LJV, Luisi BF. 2018. Multidrug efflux pumps: structure, function and regulation. Nat Rev Microbiol 16:523–539. doi:10.1038/s41579-018-0048-6 30002505

[8] Pacheco JO, Alvarez-ortega C, Rico MA. 2017. Metabolic compensation of fitness costs is a general outcome for antibiotic- mutants overexpressing efflux pumps. MBio 8:1. doi:10.1128/mBio.00500-17 PMC5527304 28743808

[9] Wood SJ, Kuzel TM, Shafikhani SH. 2023. Pseudomonas aeruginosa: infections, animal modeling, and therapeutics. Cells 12:199. doi:10.3390/cells12010199 36611992 PMC9818774

[10] Elfadadny A, Ragab RF, AlHarbi M, Badshah F, Ibáñez-Arancibia E, Farag A, Hendawy AO, De Los Ríos-Escalante PR, Aboubakr M, Zakai SA, Nageeb WM. 2024. Antimicrobial resistance of Pseudomonas aeruginosa: navigating clinical impacts, current resistance trends, and innovations in breaking therapies. Front Microbiol 15:1374466. doi:10.3389/fmicb.2024.1374466 38646632 PMC11026690

[11] Butler MS, Gigante V, Sati H, Paulin S, Al-Sulaiman L, Rex JH, Fernandes P, Arias CA, Paul M, Thwaites GE, Czaplewski L, Alm RA, Lienhardt C, Spigelman M, Silver LL, Ohmagari N, Kozlov R, Harbarth S, Beyer P. 2022. Analysis of the clinical pipeline of treatments for drug-resistant bacterial infections: despite progress, more action is needed. Antimicrob Agents Chemother 66:e0199121. doi:10.1128/AAC.01991-21 35007139 PMC8923189

[12] Murugaiyan J, Kumar PA, Rao GS, Iskandar K, Hawser S, Hays JP, Mohsen Y, Adukkadukkam S, Awuah WA, Jose RAM, Sylvia N, Nansubuga EP, Tilocca B, Roncada P, Roson-Calero N, Moreno-Morales J, Amin R, Kumar BK, Kumar A, Toufik A-R, Zaw TN, Akinwotu OO, Satyaseela MP, van Dongen MBM. 2022. Progress in alternative strategies to combat antimicrobial resistance: focus on antibiotics. Antibiotics (Basel) 11:1. doi:10.3390/antibiotics11020200 PMC8868457 35203804

[13] Roson-Calero N, Ballesté-Delpierre C, Fernández J, Vila J. 2023. Insights on current strategies to decolonize the gut from multidrug-resistant bacteria: pros and cons. Antibiotics (Basel) 12:1–13. doi:10.3390/antibiotics12061074 PMC10295446 37370393

[14] Dhanda G, Mukherjee R, Basak D, Haldar J. 2022. Small-molecular adjuvants with weak membrane perturbation potentiate antibiotics against Gram-negative superbugs. ACS Infect Dis 8:1086–1097. doi:10.1021/acsinfecdis.2c00092 35404568

[15] Dey R, Mukherjee S, Mukherjee R, Haldar J. 2023. Small molecular adjuvants repurpose antibiotics towards Gram-negative bacterial infections and multispecies bacterial biofilms. Chem Sci 15:259–270. doi:10.1039/D3SC05124B 38143555 PMC10739173

[16] Wright GD. 2016. Antibiotic adjuvants: rescuing antibiotics from resistance. Trends Microbiol 24:862–871. doi:10.1016/j.tim.2016.06.009 27430191

[17] Fratoni AJ, Berry AV, Liu X, Chen X, Wu Y, Nicolau DP, Abdelraouf K. 2023. Imipenem/funobactam (formerly XNW4107) in vivo pharmacodynamics against serine carbapenemase-producing Gram-negative bacteria: a novel modelling approach for time-dependent killing. J Antimicrob Chemother 78:2343–2353. doi:10.1093/jac/dkad242 37667103 PMC10477119

[18] Li Y, Yan M, Xue F, Zhong W, Liu X, Chen X, Wu Y, Zhang J, Wang Q, Zheng B, Lv Y. 2022. In vitro and in vivo activities of a novel β-lactamase inhibitor combination imipenem/XNW4107 against recent clinical Gram-negative bacilli from China. J Glob Antimicrob Resist 31:1–9. doi:10.1016/j.jgar.2022.07.006 35820591

[19] Corbett D, Wise A, Langley T, Skinner K, Trimby E, Birchall S, Dorali A, Sandiford S, Williams J, Warn P, Vaara M, Lister T. 2017. Potentiation of antibiotic activity by a novel cationic peptide: potency and spectrum of activity of SPR741. Antimicrob Agents Chemother 61:1–10. doi:10.1128/AAC.00200-17 PMC5527571 28533232

[20] Vaara M, Porro M. 1996. Group of peptides that act synergistically with hydrophobic antibiotics against gram-negative enteric bacteria. Antimicrob Agents Chemother 40:1801–1805. doi:10.1128/AAC.40.8.1801 8843284 PMC163420

[21] Bahar AA, Ren D. 2013. Antimicrobial peptides. Pharmaceuticals (Basel) 6:1543–1575. doi:10.3390/ph6121543 24287494 PMC3873676

[22] Hancock REW. 1997. Peptide antibiotics. Lancet 349:418–422. doi:10.1016/S0140-6736(97)80051-7 9033483

[23] Solé M, Fàbrega A, Cobos-Trigueros N, Zamorano L, Ferrer-Navarro M, Ballesté-Delpierre C, Reustle A, Castro P, Nicolás JM, Oliver A, Martínez JA, Vila J. 2015. In vivo evolution of resistance of Pseudomonas aeruginosa strains isolated from patients admitted to an intensive care unit: mechanisms of resistance and antimicrobial exposure. J Antimicrob Chemother 70:3004–3013. doi:10.1093/jac/dkv228 26260130

[24] Mulet X, Moyá B, Juan C, Macià MD, Pérez JL, Blázquez J, Oliver A. 2011. Antagonistic interactions of Pseudomonas aeruginosa antibiotic resistance mechanisms in planktonic but not biofilm growth. Antimicrob Agents Chemother 55:4560–4568. doi:10.1128/AAC.00519-11 21807976 PMC3186965

[25] Stover CK, Pham XQ, Erwin AL, Mizoguchi SD, Warrener P, Hickey MJ, Brinkman FS, Hufnagle WO, Kowalik DJ, Lagrou M, et al.. 2000. Complete genome sequence of Pseudomonas aeruginosa PAO1, an opportunistic pathogen. Nature 406:959–964. doi:10.1038/35023079 10984043

[26] Held K, Ramage E, Jacobs M, Gallagher L, Manoil C. 2012. Sequence-verified two-allele transposon mutant library for Pseudomonas aeruginosa PAO1. J Bacteriol 194:6387–6389. doi:10.1128/JB.01479-12 22984262 PMC3497512

[27] Standards P, Testing AS. 2020. M100 Performance standards for antimicrobial clinical and laboratory standards institute

[28] European Committee forAntimicrobial SusceptibilityTesting (EUCAST). 2000. Terminology relating to methods for the determination of susceptibility of bacteria to antimicrobial agents. Clin Microbiol Infect 6:503–508. doi:10.1046/j.1469-0691.2000.00149.x 11168186

[29] Langmead B, Salzberg SL. 2012. Fast gapped-read alignment with Bowtie 2. Nat Methods 9:357–359. doi:10.1038/nmeth.1923 22388286 PMC3322381

[30] Li H, Handsaker B, Wysoker A, Fennell T, Ruan J, Homer N, Marth G, Abecasis G, Durbin R, 1000 Genome Project Data Processing Subgroup. 2009. The sequence alignment/map format and SAMtools. Bioinformatics 25:2078–2079. doi:10.1093/bioinformatics/btp352 19505943 PMC2723002

[31] DePristo MA, Banks E, Poplin R, Garimella KV, Maguire JR, Hartl C, Philippakis AA, del Angel G, Rivas MA, Hanna M, McKenna A, Fennell TJ, Kernytsky AM, Sivachenko AY, Cibulskis K, Gabriel SB, Altshuler D, Daly MJ. 2011. A framework for variation discovery and genotyping using next-generation DNA sequencing data. Nat Genet 43:491–498. doi:10.1038/ng.806 21478889 PMC3083463

[32] Gomis-Font MA, Cabot G, López-Argüello S, Zamorano L, Juan C, Moyá B, Oliver A. 2022. Comparative analysis of in vitro dynamics and mechanisms of ceftolozane/tazobactam and imipenem/relebactam resistance development in Pseudomonas aeruginosa XDR high-risk clones. J Antimicrob Chemother 77:957–968. doi:10.1093/jac/dkab496 35084040

[33] Cingolani P, Platts A, Wang LL, Coon M, Nguyen T, Wang L, Land SJ, Lu X, Ruden DM. 2012. A program for annotating and predicting the effects of single nucleotide polymorphisms, SnpEff: SNPs in the genome of Drosophila melanogaster strain w^1118^; iso-2; iso-3. Fly (Austin) - FLY 6:80–92. doi:10.4161/fly.19695 22728672 PMC3679285

[34] Uppu DSSM, Manjunath GB, Yarlagadda V, Kaviyil JE, Ravikumar R, Paramanandham K, Shome BR, Haldar J. 2015. Membrane-active macromolecules resensitize NDM-1 Gram-negative clinical isolates to tetracycline antibiotics. PLoS ONE 10:e0119422. doi:10.1371/journal.pone.0119422 25789871 PMC4366164

[35] Breidenstein EBM, de la Fuente-Núñez C, Hancock REW. 2011. Pseudomonas aeruginosa: all roads lead to resistance. Trends Microbiol 19:419–426. doi:10.1016/j.tim.2011.04.005 21664819

[36] Lambert PA. 2002. Mechanisms of antibiotic resistance in Pseudomonas aeruginosa. J R Soc Med 95:22–26. 12216271 PMC1308633

[37] Pang Z, Raudonis R, Glick BR, Lin T-J, Cheng Z. 2019. Antibiotic resistance in Pseudomonas aeruginosa: mechanisms and alternative therapeutic strategies. Biotechnol Adv 37:177–192. doi:10.1016/j.biotechadv.2018.11.013 30500353

[38] Zavascki AP, Goldani LZ, Li J, Nation RL. 2007. Polymyxin B for the treatment of multidrug-resistant pathogens: a critical review. J Antimicrob Chemother 60:1206–1215. doi:10.1093/jac/dkm357 17878146

[39] Song M, Liu Y, Huang X, Ding S, Wang Y, Shen J, Zhu K. 2020. A broad-spectrum antibiotic adjuvant reverses multidrug-resistant Gram-negative pathogens. Nat Microbiol 5:1040–1050. doi:10.1038/s41564-020-0723-z 32424338

[40] MacNair CR, Farha MA, Serrano-Wu MH, Lee KK, Hubbard B, Côté J-P, Carfrae LA, Tu MM, Gaulin JL, Hunt DK, Hung DT, Brown ED. 2022. Preclinical development of pentamidine analogs identifies a potent and nontoxic antibiotic adjuvant. ACS Infect Dis 8:768–777. doi:10.1021/acsinfecdis.1c00482 35319198

[41] Gill EE, Franco OL, Hancock REW, Catolica U, Bosco D, Biotecnologia P, Grande C. 2015. Antibiotic adjuvants: diverse strategies for controlling drug-resistant pathogens. Chem Biol Drug Des 85:56–78. doi:10.1111/cbdd.12478 25393203 PMC4279029

[42] Hancock REW, Wong PGW. 1984. Compounds which increase the permeability of the Pseudomonas aeruginosa outer membrane. Antimicrob Agents Chemother 26:48–52. doi:10.1128/AAC.26.1.48 6433788 PMC179915

[43] Abraham N, Kwon DH. 2009. A single amino acid substitution in PmrB is associated with polymyxin B resistance in clinical isolate of Pseudomonas aeruginosa. FEMS Microbiol Lett 298:249–254. doi:10.1111/j.1574-6968.2009.01720.x 19663916

[44] Lee J-Y, Ko KS. 2014. Mutations and expression of PmrAB and PhoPQ related with colistin resistance in Pseudomonas aeruginosa clinical isolates. Diagn Microbiol Infect Dis 78:271–276. doi:10.1016/j.diagmicrobio.2013.11.027 24412662

[45] Marano V, Marascio N, Pavia G, Lamberti AG, Quirino A, Musarella R, Casalinuovo F, Mazzitelli M, Trecarichi EM, Torti C, Matera G, Liberto MC. 2020. Identification of pmrB mutations as putative mechanism for colistin resistance in A. baumannii strains isolated after in vivo colistin exposure. Microb Pathog 142:104058. doi:10.1016/j.micpath.2020.104058 32058026

[46] Lorusso AB, Carrara JA, Barroso CDN, Tuon FF, Faoro H. 2022. Role of efflux pumps on antimicrobial resistance in Pseudomonas aeruginosa. Int J Mol Sci 23:15779. doi:10.3390/ijms232415779 36555423 PMC9779380

[47] Lomovskaya O, Warren MS, Lee A, Galazzo J, Fronko R, Lee MAY, Blais J, Cho D, Chamberland S, Renau TOM, Leger R, Hecker S, Watkins W, Hoshino K, Ishida H, Lee VJ. 2001. Identification and characterization of inhibitors of multidrug resistance efflux pumps in Pseudomonas aeruginosa: novel agents for combination therapy. Antimicrob Agents Chemother 45:105–116. doi:10.1128/AAC.45.1.105-116.2001 11120952 PMC90247

[48] Stocks SM. 2004. Mechanism and use of the commercially available viability stain, BacLight. Cytometry A 61:189–195. doi:10.1002/cyto.a.20069 15382024

[49] Sutcliffe JA, O’Brien W, Fyfe C, Grossman TH. 2013. Antibacterial activity of eravacycline (TP-434), a novel fluorocycline, against hospital and community pathogens. Antimicrob Agents Chemother 57:5548–5558. doi:10.1128/AAC.01288-13 23979750 PMC3811277

[50] Zhanel GG, Cheung D, Adam H, Zelenitsky S, Golden A, Schweizer F, Gorityala B, Lagacé-Wiens PRS, Walkty A, Gin AS, Hoban DJ, Karlowsky JA. 2016. Review of eravacycline, a novel fluorocycline antibacterial agent. Drugs (Abingdon Engl) 76:567–588. doi:10.1007/s40265-016-0545-8 26863149

[51] Brown DFJ, Canton R, MacGowan AP, Mouton JW, Rodloff A, Goldstein F, Odenholt I, Steinbakk M, Varaldo P, Hryniewicz W, Kahlmeter G, The European Committee on Antimicrobial Susceptibility Testing (EUCAST) Steering Committee. 2006. EUCAST technical note on tigecycline. Clin Microbiol Infect 12:1147–1149. doi:10.1111/j.1469-0691.2006.01578.x 16700696

[52] Jones RN, Wilson ML, Weinstein MP, Stilwell MG, Mendes RE. 2013. Contemporary potencies of minocycline and tetracycline HCL tested against Gram-positive pathogens: SENTRY Program results using CLSI and EUCAST breakpoint criteria. Diagn Microbiol Infect Dis 75:402–405. doi:10.1016/j.diagmicrobio.2013.01.022 23514756

[53] Dallas SD, McGee L, Limbago B, Patel JB, McElmeel ML, Fulcher LC, Lonsway DR, Jorgensen JH. 2013. Development of doxycycline MIC and disk diffusion interpretive breakpoints and revision of tetracycline breakpoints for Streptococcus pneumoniae. J Clin Microbiol 51:1798–1802. doi:10.1128/JCM.00125-13 23554197 PMC3716062

[54] Asadi A, Abdi M, Kouhsari E, Panahi P, Sholeh M, Sadeghifard N, Amiriani T, Ahmadi A, Maleki A, Gholami M. 2020. Minocycline, focus on mechanisms of resistance, antibacterial activity, and clinical effectiveness: back to the future. J Glob Antimicrob Resist 22:161–174. doi:10.1016/j.jgar.2020.01.022 32061815

[55] Brennan-Krohn T, Grote A, Rodriguez S, Kirby JE, Earl AM. 2022. Transcriptomics reveals how minocycline-colistin synergy overcomes antibiotic resistance in multidrug-resistant Klebsiella pneumoniae. Antimicrob Agents Chemother 66:e0196921. doi:10.1128/aac.01969-21 35041511 PMC8923212

[56] Rajakani SG, Xavier BB, Sey A, Mariem EB, Lammens C, Goossens H, Glupczynski Y, Malhotra-Kumar S. 2023. Insight into antibiotic synergy combinations for eliminating colistin heteroresistant Klebsiella pneumoniae. Genes (Basel) 14:1426. doi:10.3390/genes14071426 37510330 PMC10378790

[57] Jenssen H, Aspmo SI. 2008. Serum stability of peptides, p 177–186. In Peptide-based drug design. Methods in molecular biology. Humana Press, Totowa, NJ. 10.1007/978-1-59745-419-3_10 18726574

[58] Knappe D, Henklein P, Hoffmann R, Hilpert K. 2010. Easy strategy to protect antimicrobial peptides from fast degradation in serum. Antimicrob Agents Chemother 54:4003–4005. doi:10.1128/AAC.00300-10 20585128 PMC2934954

