# Supplementary material for: In vitro potentiation of tetracyclines in Pseudomonas aeruginosa by RW01, a new cyclic peptide
Source: Antimicrob Agents Chemother. 2024 Dec 23;69(2):e01459-24. doi: 10.1128/aac.01459-24 (PMC11823630; doi:10.1128/aac.01459-24)
Supplement: Supplemental material — Synthesis of RW01 peptide analogues, and flow cytometry analysis. [file aac.01459-24-s0001.pdf]

SUPPLEMENTAL MATERIAL: Bioavailability potentiation of tetracyclines in Pseudomonas aeruginosa by RW01, a new cyclic peptide.

SYNTHESIS OF RW01 PEPTIDE ANALOGUES

Albert Ruiz-Soriano, Xavier Just-Baringo

Laboratori de Química Orgànica, Facultat de Farmàcia, Universitat de Barcelona, 08028 Barcelona (Spain)

e-mail: xavier.just@ub.edu

1. General Information

All solvents and reagents used were purchased from commercial suppliers and used without further purification. ^{1}H-NMR spectra were obtained at room temperature on a Bruker 400 MHz spectrometer. ^{13}C-NMR spectra were obtained at 100 MHz. All NMR spectra were processed using MestReNova NMR software. Chemical shifts are reported in parts per million (ppm) and coupling constants (*J*) are reported in Hz. Splitting patterns are reported as follows: singlet (s), doublet (d), triplet (t), quadruplet (q), quintuplet (quint), doublet of doublets (dd), doublet of doublets of doublets (ddd), multiplet (m), etc. NMR signals were assigned using the appropriate 2D NMR experiments (*i.e.* HSQC and HMBC when necessary). High-resolution mass spectrometry (HRMS) was performed by Unitat de Cromatografia de Gasos-Espectrometria de Masses Aplicada, Centres Científics i Tecnològics de la Univeristat de Barcelona (CCiTUB).

2. Peptide Analysis and Purification

2.1. HPLC Analysis

HPLC analysis was performed on a Waters Alliance 2695 separation module coupled to a Waters 2996 photodiode detector (PDA) and to an electrospray ionization source Waters ACQUITY QDa detector, using the MassLynx 4.1 software for data acquisition and a XSelect CSH C18 OBD column. The flow rate was 0.6 mL·min^{-1}, and MeCN (0.1% formic acid) and H_2O (0.1% formic acid) were used as solvents. The elution runtime was 3.5 min at 50 °C.

2.2. Reserve Phase Purification

Reverse phase purification was performed on a Biotage Isolera One preparative purification system using Biotage Sfär C18 D-Duo 100 Å 30 □m (6 g) columns. The flow rate was 6 mL·min^{-1}, and MeCN (0.1% TFA) and H_2O (0.1% TFA) were used as solvents.

3. Synthesis: Experimental Details and Characterization

S1. 2-(4-Benzoylphenoxy)acetic acid[1]

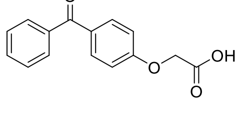

[1] Kappel, J. C.; Fan, Y. C.; Lam, K. S. *J. Comb. Chem.* **2008**, 10, 333

A solution of 4-hydroxybenzophenone (1.00 g, 5.04 mmol), bromoacetic acid (1.39 g, 10.0 mmol), and anhydrous K_2CO_3 (4.20 g, 30.2 mmol) in dry acetone (39 mL) was stirred at reflux. After 15 h, it was allowed to cool to room temperature before removing volatiles under vacuum. The solid residue was taken up in a mixture of H_2O (280 mL) and concentrated aqueous dibasic sodium phosphate (28 mL), dissolved with heating and then acidified to pH 1 with 6 N HCl. After the mixture was cooled in an ice bath, the precipitate was collected, washed with water and dried under vacuum in the presence of P_2O_5 providing 2-(4-benzoylphenoxy)acetic acid as a white powder (1.21 g, 94%). The product thus obtained matched the one reported in the literature.[1]

^{1}H-NMR (400 Hz, CDCl3): δ 13.2 (brs, CO_2H, 1 H), 7.74 (d, J = 9.0 Hz, $C_{Ar}H$, 2 H), 7.71-7.62 (m, $C_{Ar}H$, 3 H), 7.55 (t, J = 7.6 Hz, $C_{Ar}H$, 2 H), 7.07 (d, J = 9.0 Hz, $C_{Ar}H$, 2 H), 4.80 (s, CH_2CO_2H, 2 H) ppm.

S2. RW01-BP

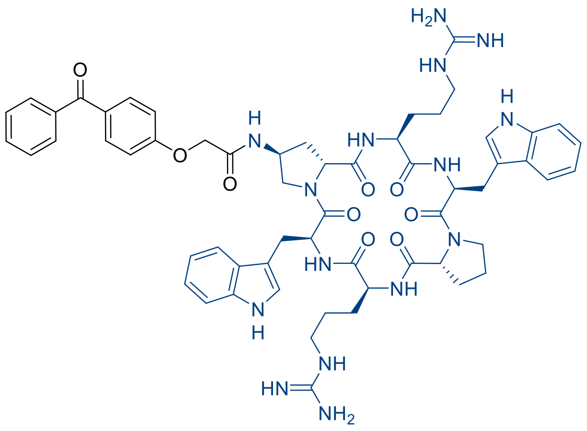

To a nitrogen-flushed vial charged with cyclic peptide **RW01** (100 mg, 0.11 mmol), 2-(4-benzoylphenoxy)acetic acid (32 mg, 0.12 mmol) and BOP (54 mg, 0.12 mmol) were added anhydrous DMF (3.3 mL) and DIPEA (178 µL, 1.0 mmol) and the resulting solution was stirred at room temperature. After 15 h, volatiles were removed under vacuum and the crude product was purified by reverse phase (0% to 50% MeCN) to yield the title product as a pale pink solid (55 mg, 43%). HRMS calcd for $C_{59}H_{71}N_{15}O_9$ $[M+2H]^{+2}$: 566.7774, found 566.7777.

S3. RW01-Glu-OH

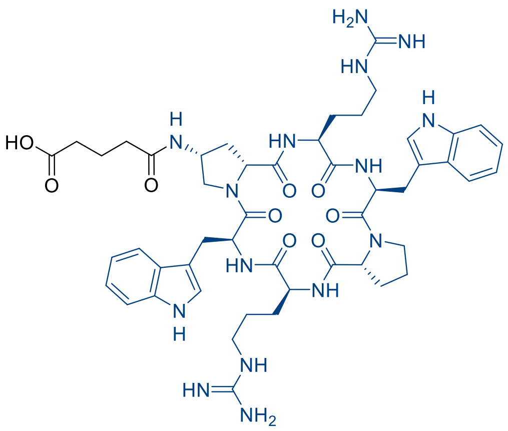

To a nitrogen-flushed tube charged with a solution of cyclic peptide **RW01** (20 mg, 0.022 mmol) and glutaric anhydride (3 mg, 0.024 mmol) in anhydrous THF (1.0 mL) was added Et_3N (9 μL, 0.066 mmol). The tube was sealed under nitrogen and the mixture was stirred at 66 °C. After 16 h, the mixture was allowed to cool to room temperature before volatiles were removed under vacuum to yield the desired product as a white solid (22 mg, quant.). HRMS calcd for $C_{49}H_{67}N_{15}O_9$ $[M+2H]^{+2}$: 504.7618, found 504.7621.

S4. RW01-Glu-OMe

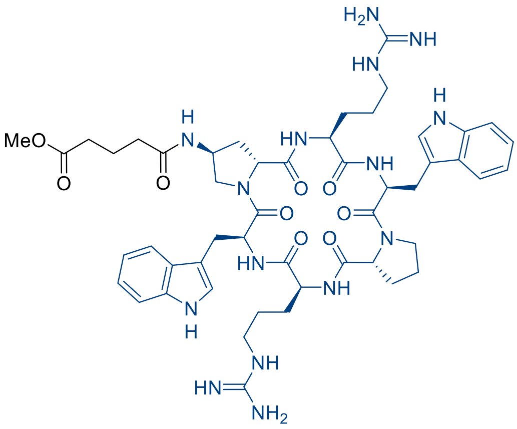

To a nitrogen-flushed tube charged with a solution of cyclic peptide **RW01** (20 mg, 0.22 mmol) in anhydrous THF (1 mL) were added glutaric acid monomethyl ester chloride (3.3 μL, 0.024 mmol) and Et_3N (9 μL, 0.066). The tube was sealed under nitrogen and the resulting mixture was stirred at 66 °C. After 16 h, the mixture was allowed to cool to room temperature before volatiles were removed under vacuum. The crude was purified by reverse phase (0% to 50% MeCN) to yield **RW01-Glu-OMe** as a white solid (10 mg, 44%). HRMS calcd for $C_{50}H_{69}N_{15}O_9$ $[M+2H]^{+2}$: 511.7696, found 511.7703.

3. NMR

2-(4-Benzoylphenoxy)acetic acid

S5. ^{1}H-NMR

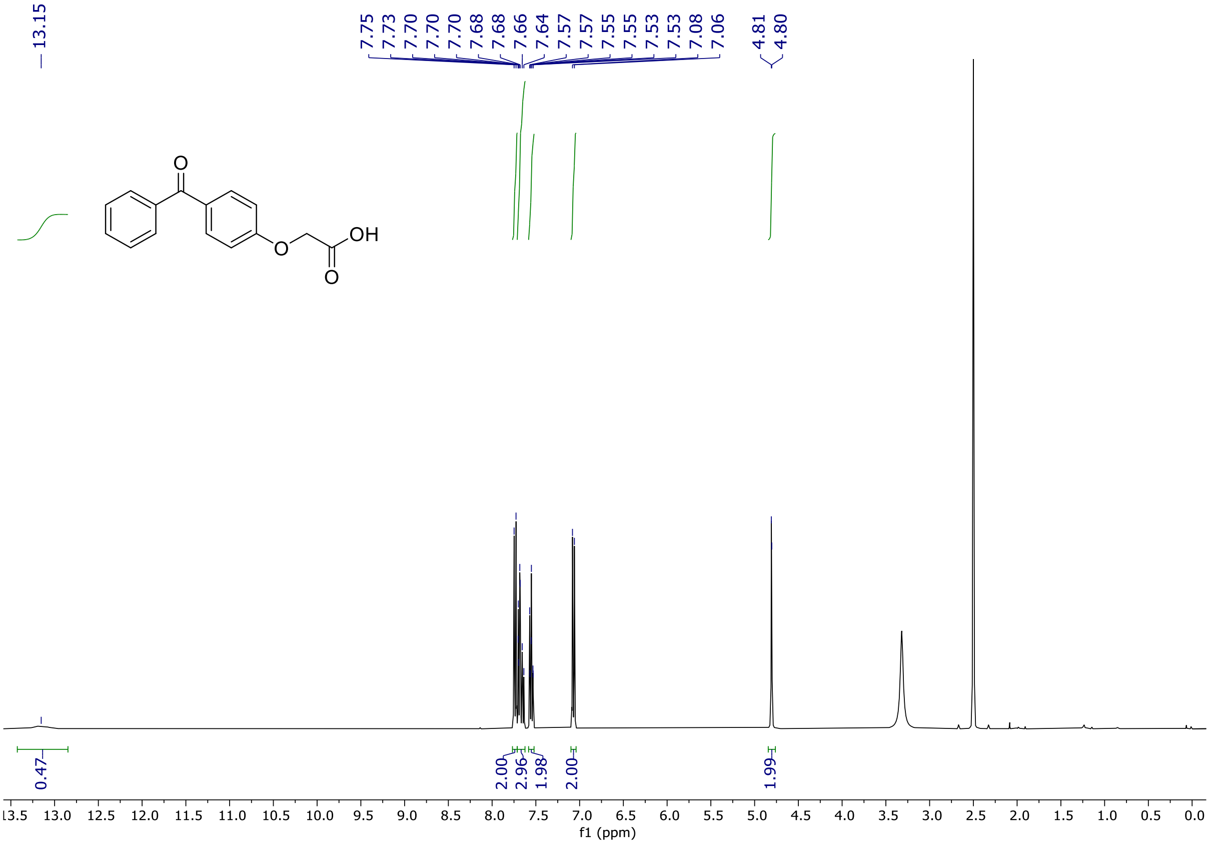

FLOW CYTOMETRY ANALYSIS

1. **General Information.** In the following figures, CP1 stands for RW01 cyclic peptide. Recounts were analysed using the FlowJo Layout. Following dot plots show the relationship between the two fluorescence markers, propidium iodide (PI) for DEAD cells and SYTO-9 for ALIVE cells. Each point represents a single cell, plotted according to its values for the two parameters.

2. **Results:**

2.1 Recounts at 4h treatment:

S6. 0’2X MIC of RW01 – 4h treatment – PAO1 wild-type strain

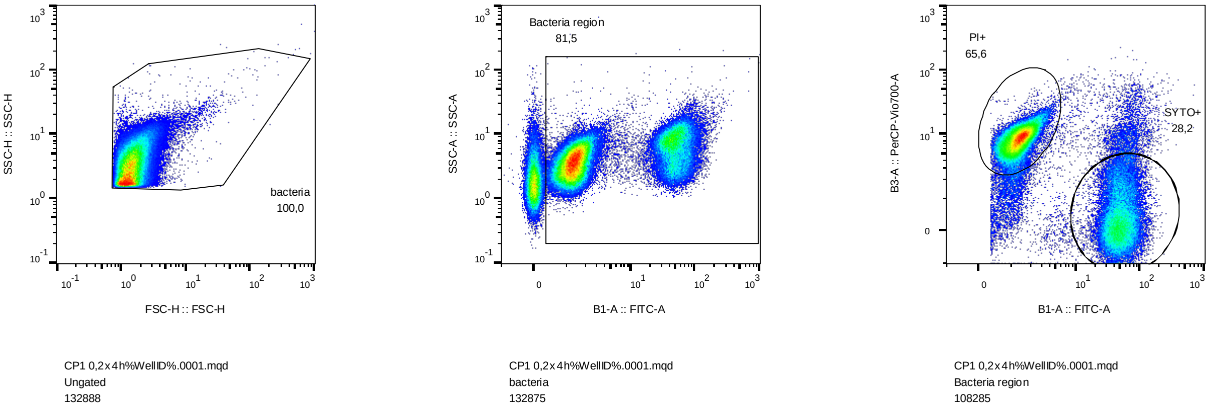

S7. 0,5X MIC of RW01 – 4h treatment – PAO1 wild-type strain

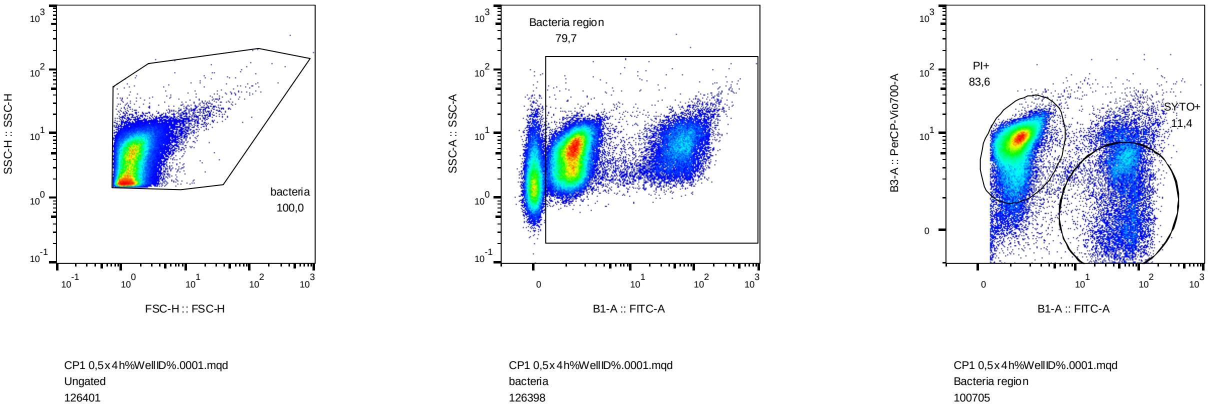

S8. 1X MIC of RW01 – 4h treatment – PAO1 wild-type strain

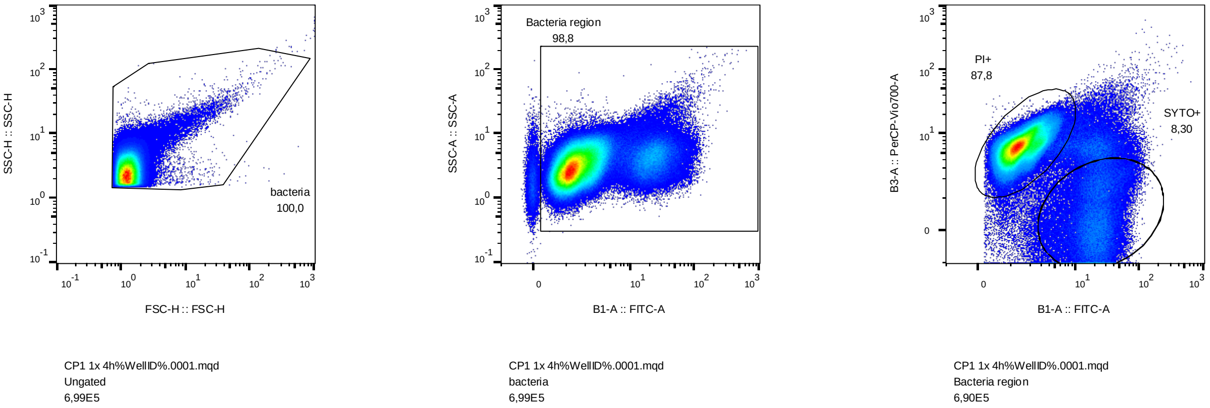

S9. 5X MIC of RW01 – 4h treatment – PAO1 wild-type strain

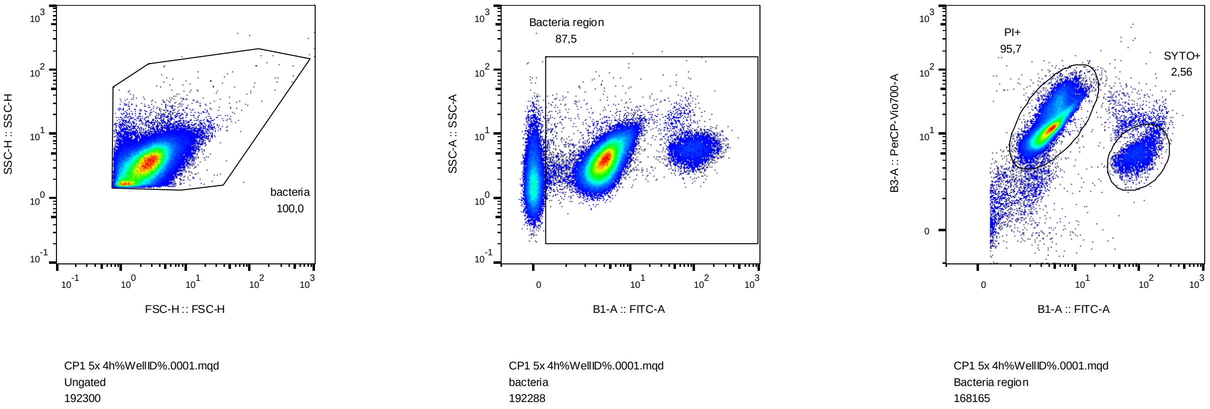

S10. 10X MIC of RW01 – 4h treatment – PAO1 wild-type strain

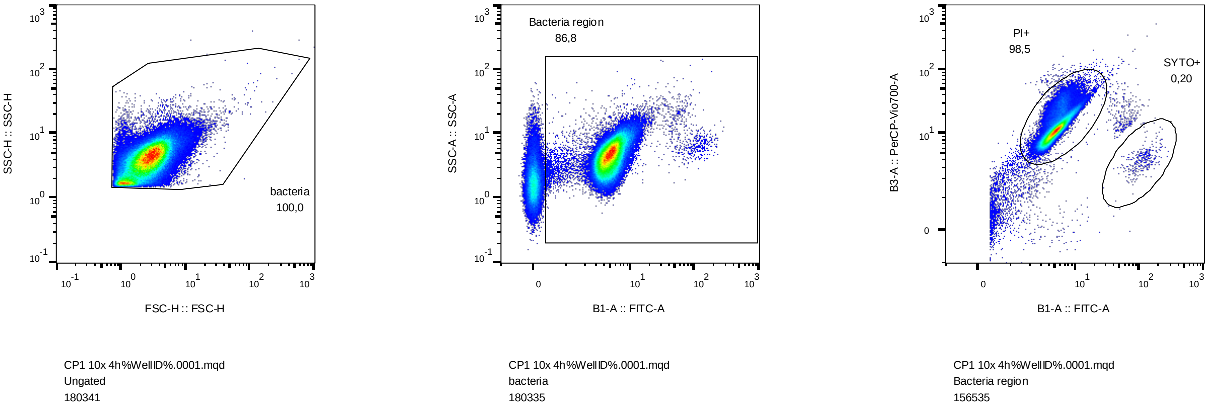

S11. 0,2X MIC of Colistin – 4h treatment – PAO1 wild-type strain

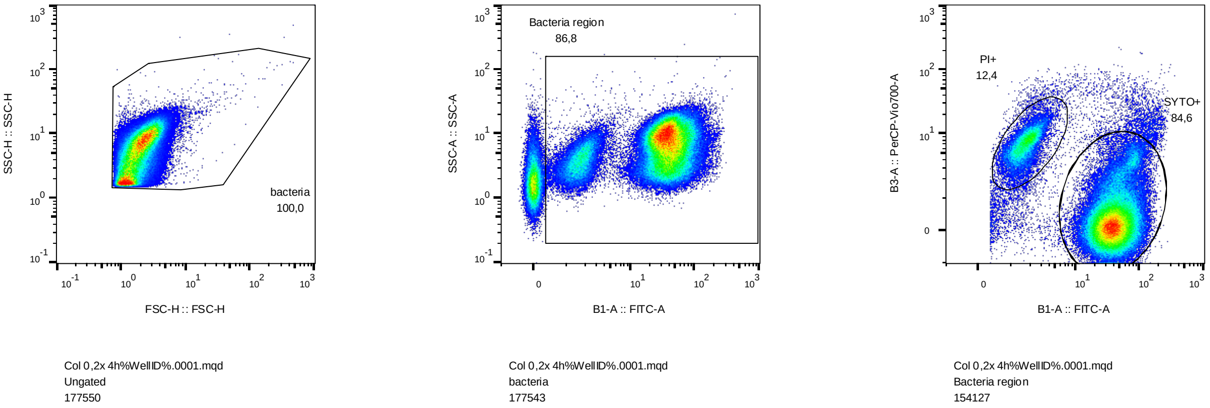

S12. 0,5X MIC of Colistin – 4h treatment – PAO1 wild-type strain

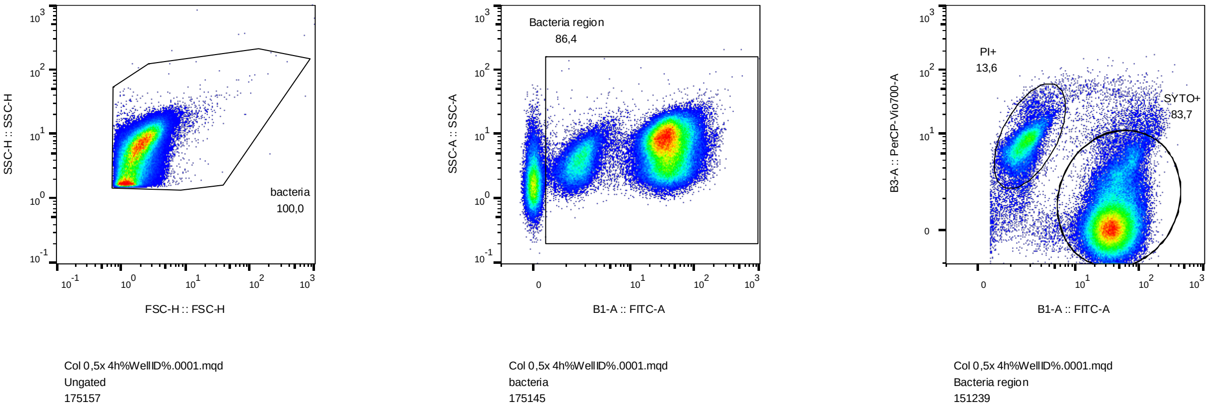

S13. 1X MIC of Colistin – 4h treatment – PAO1 wild-type strain

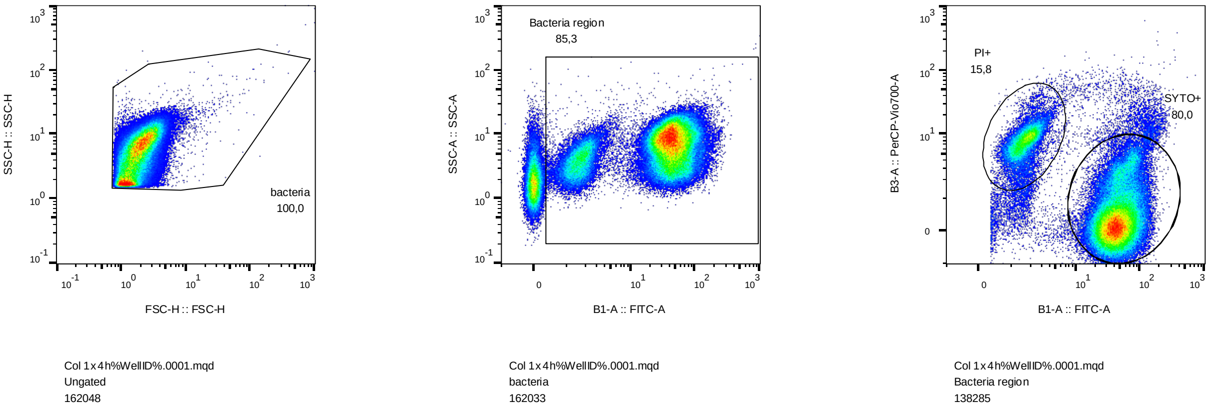

S14. 5X MIC of Colistin – 4h treatment – PAO1 wild-type strain

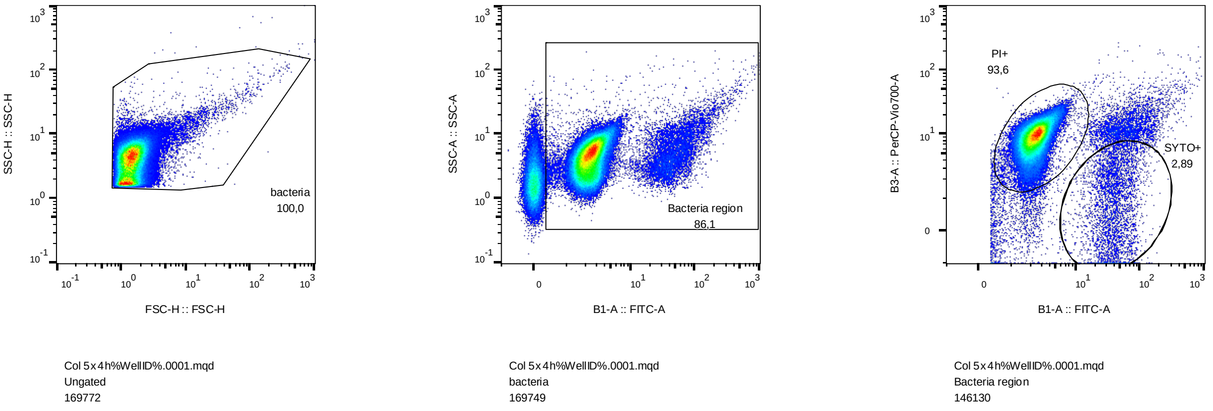

S15. 10X MIC of Colistin – 4h treatment – PAO1 wild-type strain

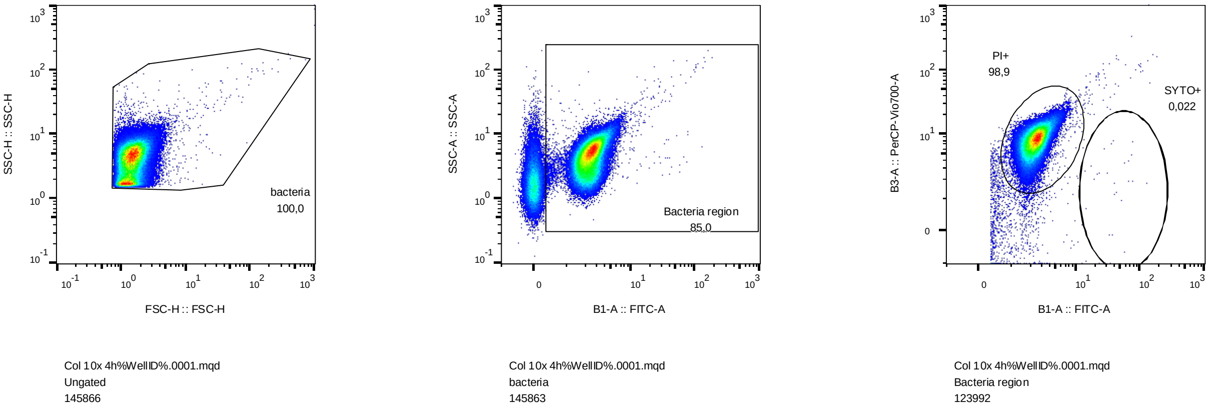

S16. No treatment – 4h PI control – PAO1 wild-type strain

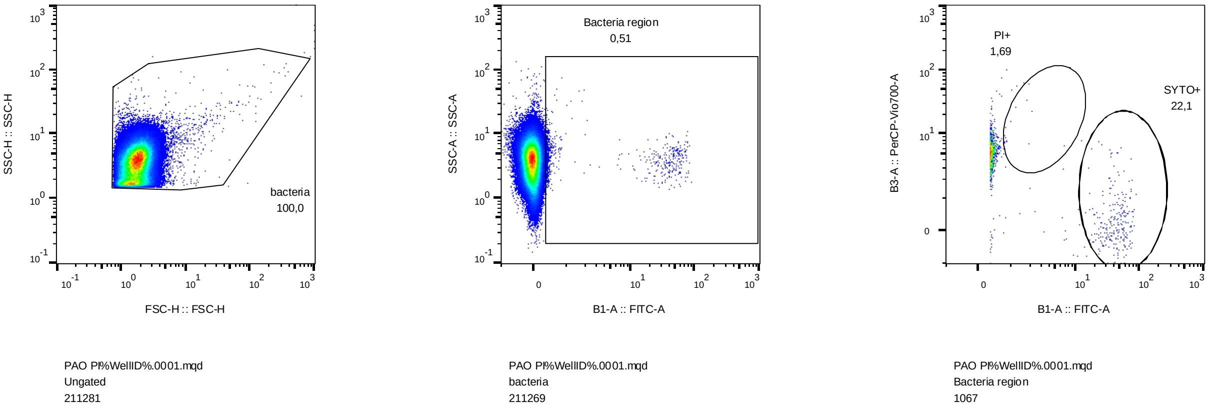

S17. No treatment – 4h SYTO-9 control – PAO1 wild-type strain

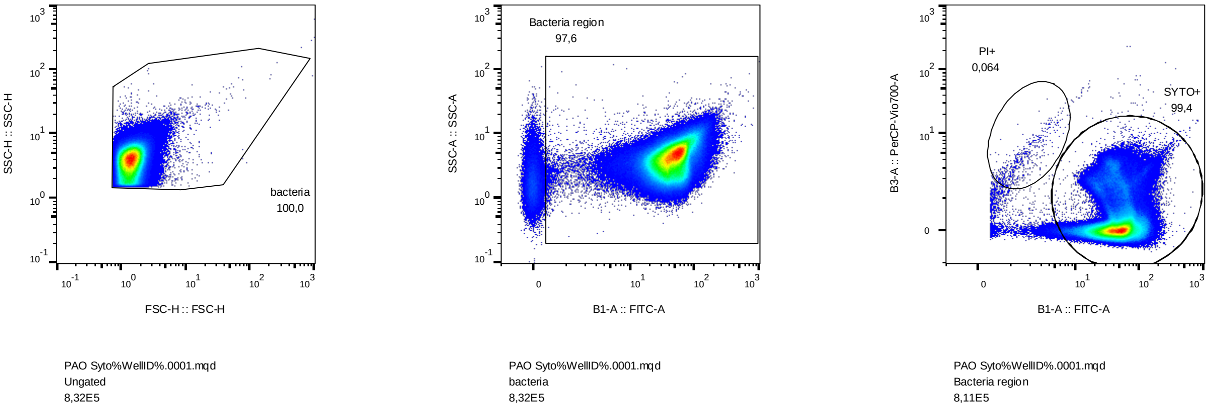

S18. No treatment – 4h no stain control – PAO1 wild-type strain

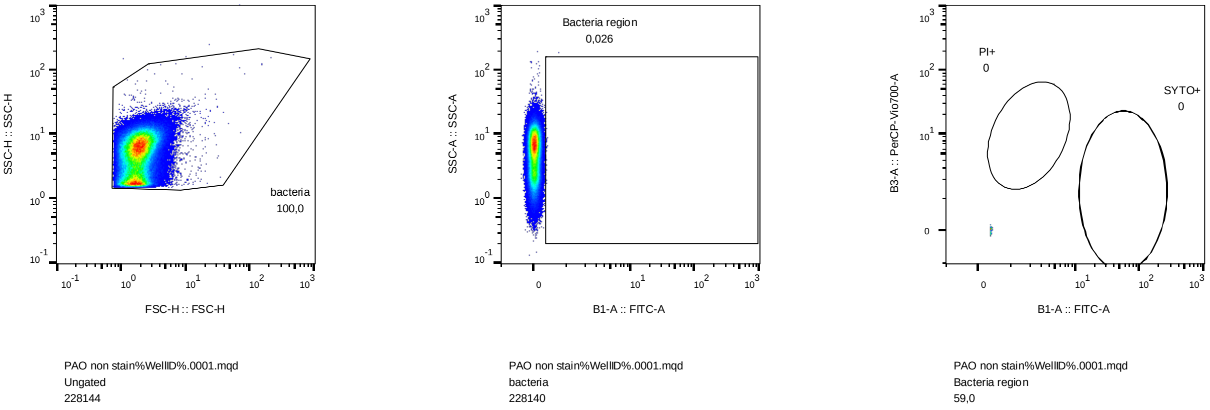

S19. Serum control – NaCl 0,9%

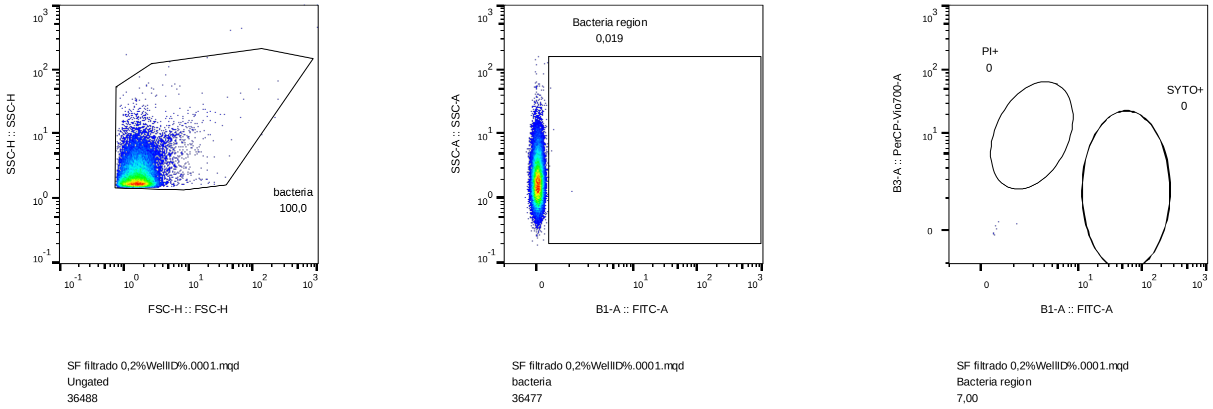

2.2 Recounts at 24h treatment:

S20. 0’2X MIC of RW01 – 24h treatment – PAO1 wild-type strain

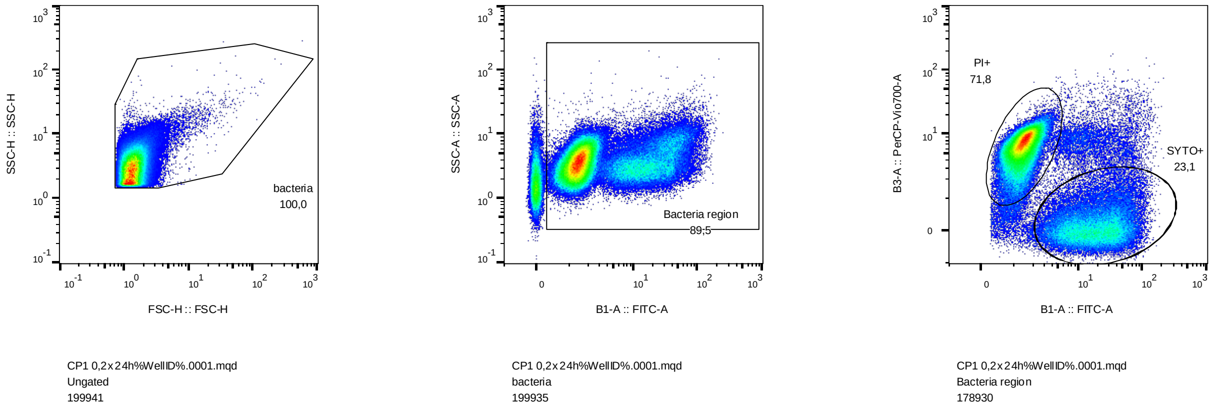

S21. 0’5X MIC of RW01 – 24h treatment – PAO1 wild-type strain

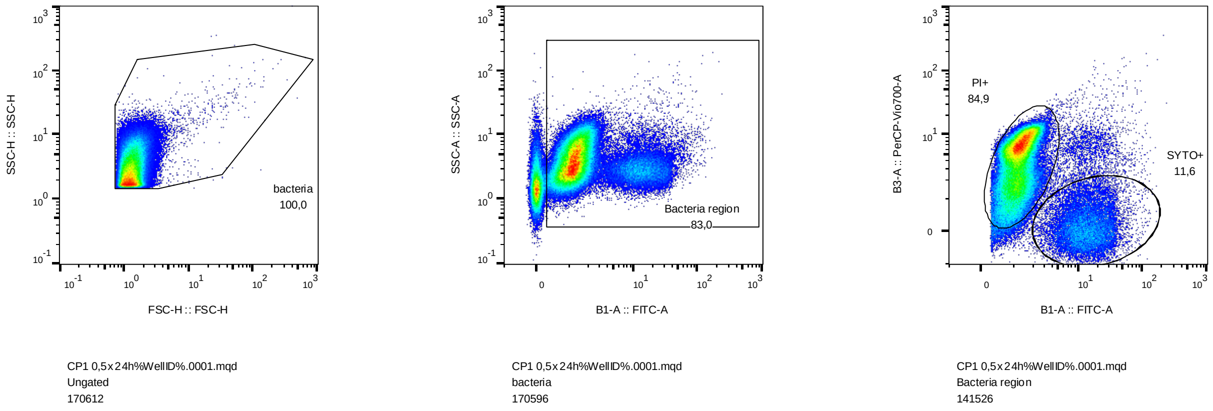

S22. 1X MIC of RW01 – 24h treatment – PAO1 wild-type strain

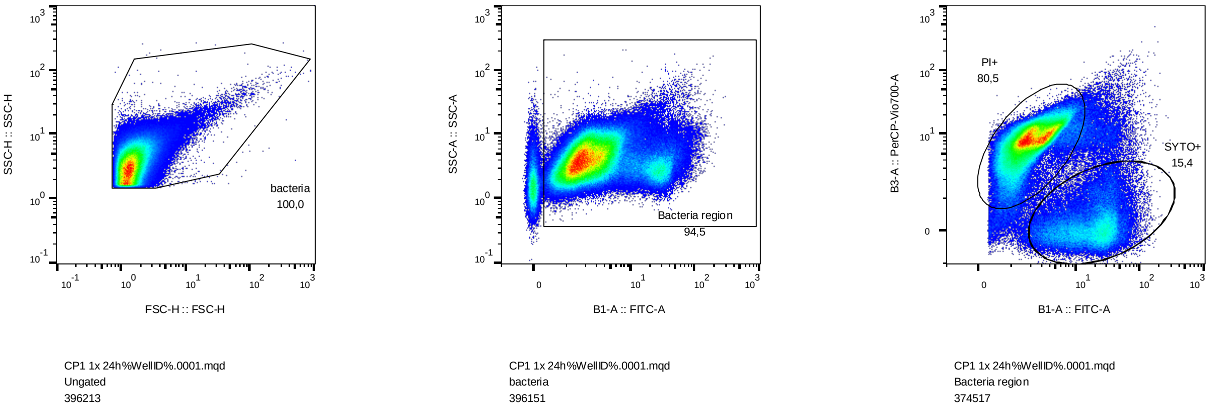

S23. 5X MIC of RW01 – 24h treatment – PAO1 wild-type strain

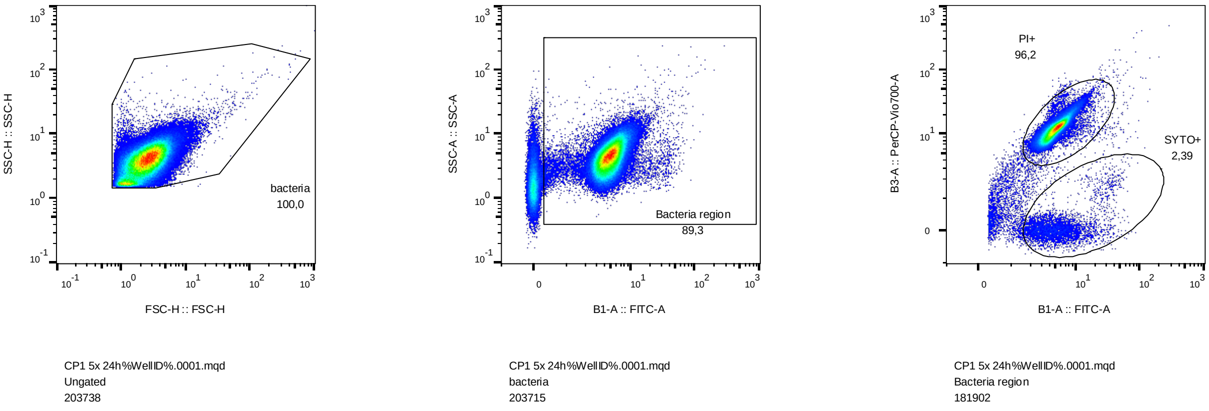

S24. 10X MIC of RW01 – 24h treatment – PAO1 wild-type strain

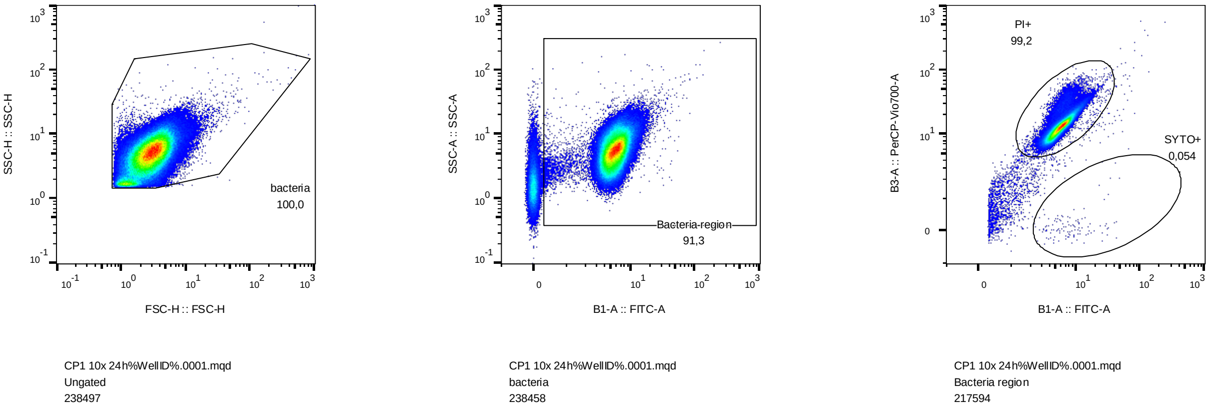

S25. 0'2X MIC of Colistin – 24h treatment – PAO1 wild-type strain

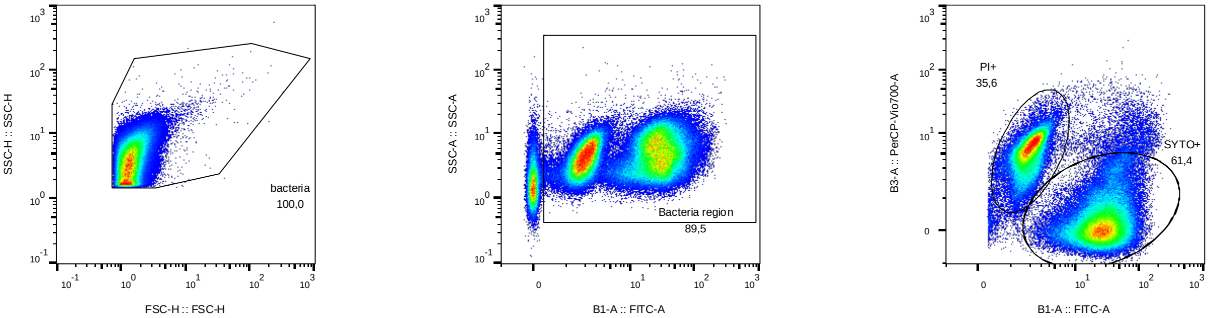

Col 0,2x 24h%WellID%.0001.mqd
Ungated
205794

Col 0,2x 24h%WellID%.0001.mqd
bacteria
205783

Col 0,2x 24h%WellID%.0001.mqd
Bacteria region
184188

S26. 0'5X MIC of Colistin – 24h treatment – PAO1 wild-type strain

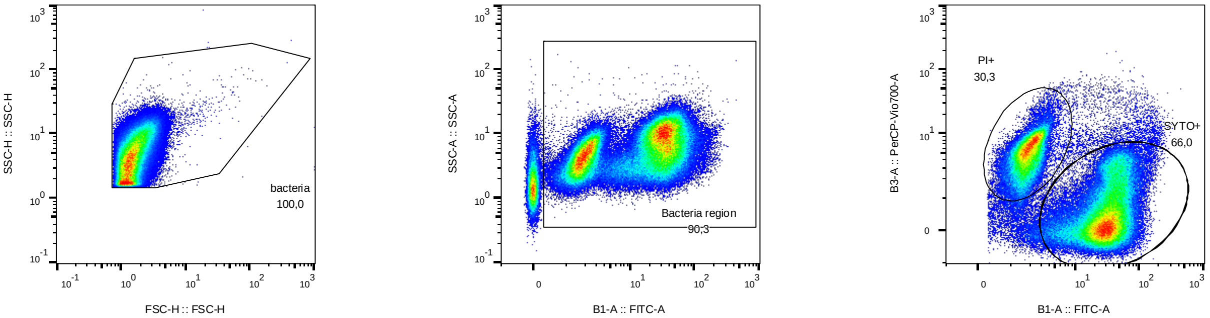

Col 0,5x 24h%WellID%.0001.mqd
Ungated
208517

Col 0,5x 24h%WellID%.0001.mqd
bacteria
208499

Col 0,5x 24h%WellID%.0001.mqd
Bacteria region
188232

S27. 1X MIC of Colistin – 24h treatment – PAO1 wild-type strain

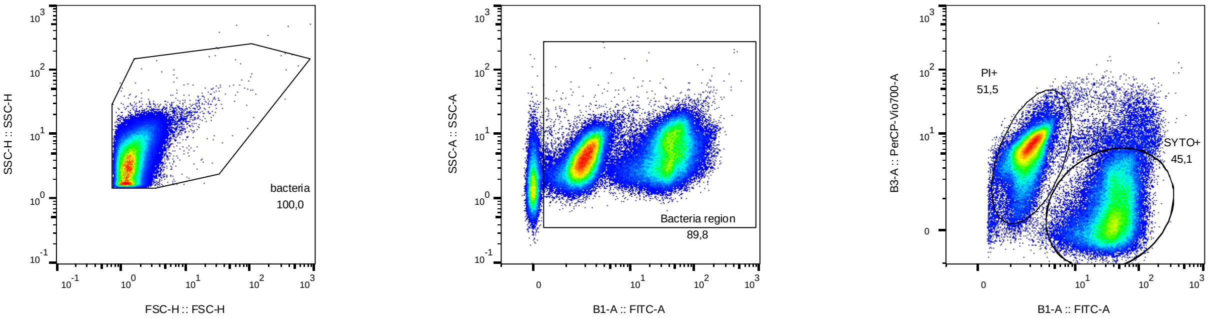

Col 1x 24h%WellID%.0001.mqd
Ungated
206633

Col 1x 24h%WellID%.0001.mqd
bacteria
206622

Col 1x 24h%WellID%.0001.mqd
Bacteria region
185487

S28. 5X MIC of Colistin – 24h treatment – PAO1 wild-type strain

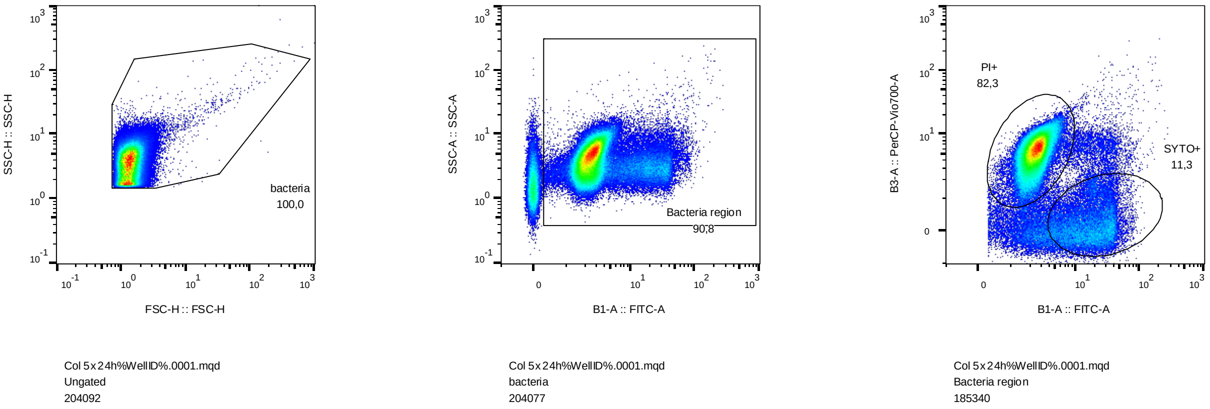

S29. 10X MIC of Colistin – 24h treatment – PAO1 wild-type strain

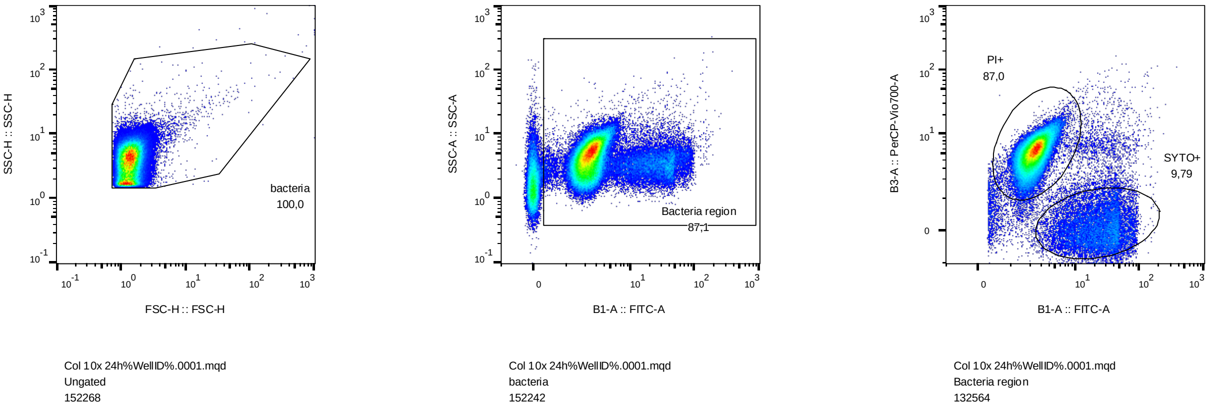

S30. No treatment – 4h PI control – PAO1 wild-type strain

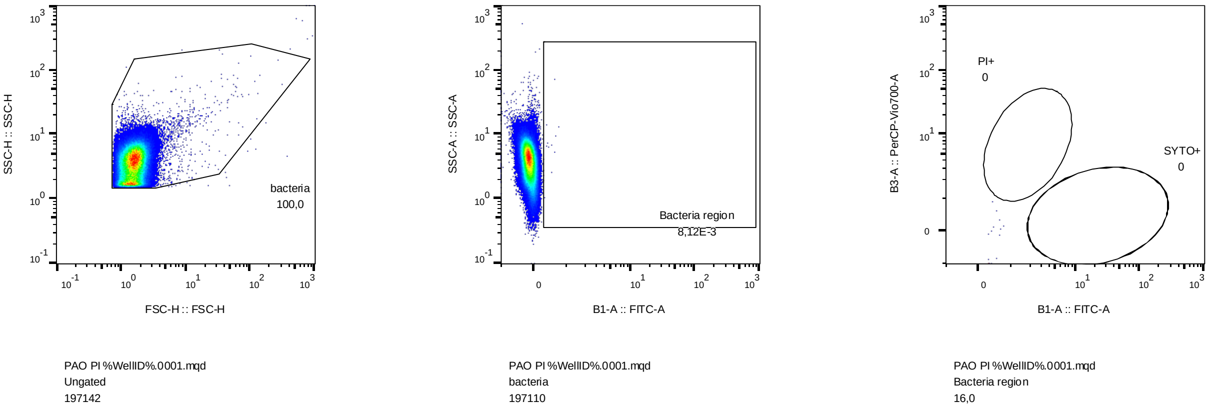

S31. No treatment – 4h SYTO-9 control – PAO1 wild-type strain

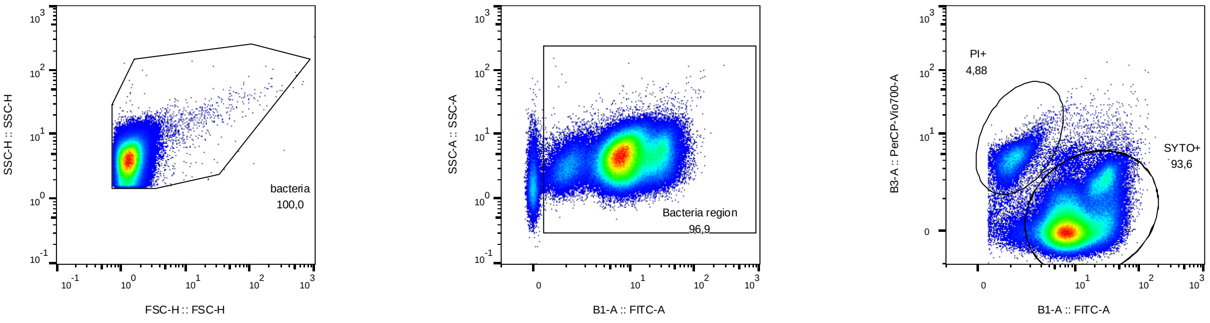

Control 24h%WellID%.0001.mqd
Ungated
340774

Control 24h%WellID%.0001.mqd
bacteria
340759

Control 24h%WellID%.0001.mqd
Bacteria region
330365

S32. Serum control – NaCl 0,9%

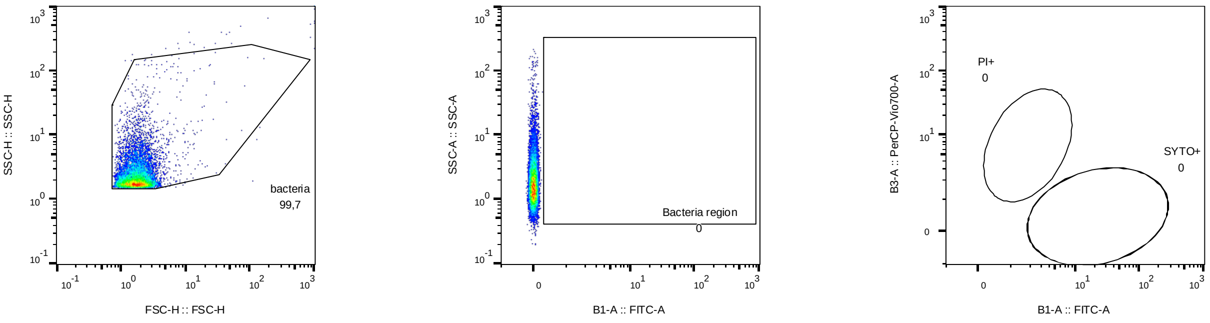

SF filtrado 0,2um%WellID%.0001.mqd
Ungated
12508

SF filtrado 0,2um%WellID%.0001.mqd
bacteria
12467

SF filtrado 0,2um%WellID%.0001.mqd
Bacteria region
0
